# *Arabidopsis thaliana* WAPL Is Essential for the Prophase Removal of Cohesin during Meiosis

**DOI:** 10.1371/journal.pgen.1004497

**Published:** 2014-07-17

**Authors:** Kuntal De, Lauren Sterle, Laura Krueger, Xiaohui Yang, Christopher A. Makaroff

**Affiliations:** Department of Chemistry and Biochemistry, Miami University, Oxford, Ohio, United States of America; Institut Jean-Pierre Bourgin, UMR1318 INRA-AgroParisTech, France

## Abstract

Sister chromatid cohesion, which is mediated by the cohesin complex, is essential for the proper segregation of chromosomes in mitosis and meiosis. The establishment of stable sister chromatid cohesion occurs during DNA replication and involves acetylation of the complex by the acetyltransferase CTF7. In higher eukaryotes, the majority of cohesin complexes are removed from chromosomes during prophase. Studies in fly and human have shown that this process involves the WAPL mediated opening of the cohesin ring at the junction between the SMC3 ATPase domain and the N-terminal domain of cohesin's α-kleisin subunit. We report here the isolation and detailed characterization of *WAPL* in *Arabidopsis thaliana*. We show that Arabidopsis contains two *WAPL* genes, which share overlapping functions. Plants in which both *WAPL* genes contain T-DNA insertions show relatively normal growth and development but exhibit a significant reduction in male and female fertility. The removal of cohesin from chromosomes during meiotic prophase is blocked in *Atwapl* mutants resulting in chromosome bridges, broken chromosomes and uneven chromosome segregation. In contrast, while subtle mitotic alterations are observed in some somatic cells, cohesin complexes appear to be removed normally. Finally, we show that mutations in *AtWAPL* suppress the lethality associated with inactivation of *AtCTF7*. Taken together our results demonstrate that WAPL plays a critical role in meiosis and raises the possibility that mechanisms involved in the prophase removal of cohesin may vary between mitosis and meiosis in plants.

## Author Summary

Wapl has been shown to play an integral role in the removal of cohesin from chromosomes during mitotic prophase. While Wapl's role appears to be conserved between yeast, fly and animal cells, structural and possible mechanistic differences have also been identified. As part of a study to better understand the protein and its role(s) we have characterized Wapl in plants. We show that *Arabidopsis* contains two copies of *WAPL* that share overlapping functions. Inactivation of the individual genes has no effect. Plants containing mutations in both genes growth normally but exhibit reduced fertility due to alterations in meiosis. Cohesin removal from chromosomes during meiotic prophase is blocked in *wapl* mutant plants resulting in unresolved bivalents and uneven chromosome segregation. In contrast, cohesin appears to be removed normally in mitotic cells. These results demonstrate that WAPL plays a critical role in removing cohesin from meiotic chromosomes. They also suggest that the mechanism involved in prophase removal of cohesin may vary between mitosis and meiosis in plants. Finally, *wapl* mutations suppress *ctf7*-associated lethality and restore normal growth and partial fertility to *ctf7* mutant plants, suggesting that sister chromatid cohesion is not essential for plant growth and development.

## Introduction

The timely establishment and dissolution of sister chromatid cohesion is essential for the proper segregation of chromosomes during cell division, as well as the repair of DNA damage and the control of transcription (reviewed in [1]–[4]). Four proteins form the core cohesin complex: Structural Maintenance of Chromosome (SMC) proteins 1 (SMC1) and 3 (SMC3), Sister Chromatid Cohesion (SSC) protein 3 (SCC3), and an α-kleisin, either SCC1 which is part of the mitotic cohesion complex, or REC8 that functions during meiosis. Studies in several organisms have shown that cohesin complex components and the general mechanisms of cohesin action are conserved across species; however variations in complex member composition and the mechanistic roles of some complex members have been observed between some species and between mitosis and meiosis (reviewed in [3], [5]–[7]).

Cohesin complexes are recruited to the chromatin by the Scc2/Scc4 complex throughout the cell cycle, with most of the complexes loaded onto chromosomes during telophase/G1 [8]–[10]. Prior to S-phase cohesin association with the chromatin is dynamic and regulated in part by a complex which has been referred to by several names, including “releasin”, the “antiestablishment” and/or the “antimaintenance” complex [11], [12]. This complex consists of the Wings apart-like protein (Wapl) and the Precocious dissociation of sisters protein 5 (Pds5) [13]–[17]. In vertebrates sororin is also part of the complex [18], [19]. The Ctf7/Eco1-dependent acetylation of SMC3 inhibits Wapl and results in the stable association of cohesin with chromosomes [20]–[24].

Cohesin is subsequently removed from chromosomes in steps [25]. While the specific details vary somewhat depending on the organism being studied, the general process appears to be relatively conserved. In higher eukaryotes, arm cohesin is removed during mitotic prophase in a Polo-like kinase, cyclin-dependent kinase and Wapl dependent process that involves opening of the cohesin ring at the junction between the SMC3 ATPase domain and the N-terminal winged-helix domain (WHD) of SCC1 [26]–[29]. Centromeric cohesin is protected by the Shugoshin (Sgo1)-protein phosphatase 2A (PP2A) complex, which binds and dephosphorylates cohesin, protecting it from Wapl [30]–[32]. At the metaphase to anaphase transition the metallo-proteinase separase is activated and cleaves the SCC1 subunit of centromere-localized cohesin, allowing the cohesin ring to open and the sister chromatids to disjoin [33]. Meiotic cohesin is removed in three steps: a prophase step, followed by the separase dependent cleavage of chromosome arm associated REC8 at anaphase I; finally centromere associated REC8 is cleaved by separase at anaphase II [34]–[36].

The importance of Wapl in controlling mitotic sister chromatid cohesion has been known for some time, but it is only recently that we have begun to understand how specifically Wapl helps facilitate the interaction of cohesin with chromosomes. Wapl was first identified in *Drosophila melanogaster* as a protein involved in the regulation of heterochromatin organization, with mutant flies containing parallel sister chromatids with loosened cohesion at their centromeres [37]. More recently structural studies on Wapl and its role(s) in sister chromatid cohesion during mitosis have been conducted in several organisms, including fungi, fly and vertebrates [38]–[40]. Wapl proteins from different species contain a conserved C-terminus with more divergent N-terminal domains. The divergent N-terminus appears to be a primary Pds5 binding region, while the C-terminus contains cohesin-binding determinants. While a number of similarities exist between the yeast and vertebrate proteins, structural and binding differences have also been identified. These results, along with the observation that *wapl* mutants in different organisms can exhibit different phenotypes, indicate that there is still much we do not understand about Wapl and how its structure is related to its function. Furthermore, while the effect of Wapl inactivation on mitosis has been studied in several organisms, little is known about the role of the protein during meiosis.

In the current study, we have characterized *WAPL* in the model organism *Arabidopsis thaliana*. We show that while AtWAPL plays a critical role in facilitating sister chromatid separation during meiosis, it appears to have a more minor role in somatic cells. *AtWAPL* mutations resulted in reduced male and female fertility but had little effect on plant growth. Meiotic defects, including alterations in chromosome structure and the separation of homologous chromosomes and sister chromatids was observed in most meiocytes. The removal of cohesin from meiotic chromosomes during prophase was blocked in *Atwapl* mutants resulting in chromosome bridges, broken chromosomes and the uneven segregation of chromosomes. Finally, we show that *AtWAPL* mutations can partially suppress the lethality associated with inactivation of the cohesin establishment factor, *AtCTF7*.

## Results

**Figure 1:**
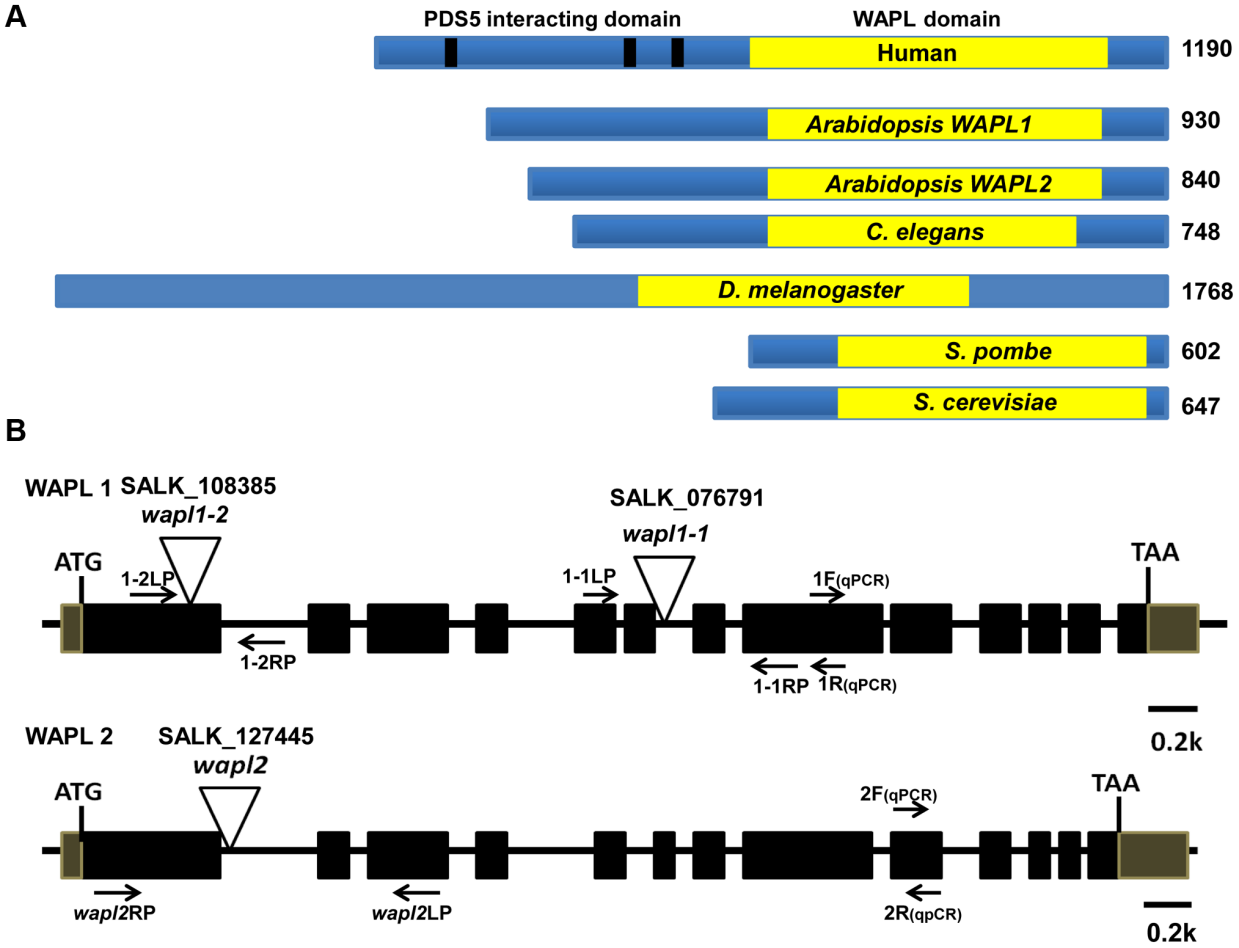
*Arabidopsis* WAPL protein and gene structures. (A) WAPL proteins from different organisms are shown. Yellow boxes represent the conserved WAPL domain. Black lines in human Wapl represent FGF motifs, which are involved in PDS5 binding. Sizes of the proteins in amino acids are shown to the right. (B) Genomic organization and T-DNA insertion sites in Arabidopsis *WAPL1* and *WAPL2*. Primer sets used for genotyping of *AtWAPL1* (1.1LP, 1.1RP and LBb1.3 for *Atwapl1-1*; 1.2LP, 1.2 RP and LBb1.3 for *Atwapl1-2*) and *AtWAPL2* (*wapl2*LP, *wapl2*RP and *LBb1.3* for *wapl2*) T-DNA lines are shown. Quantitative RT-PCR primers are indicated by 1F, 1R, 2F and 2R.

Analysis of the *Arabidopsis* genome identified two genes, which we have designated as *AtWAPL1* (At1g11060) and *AtWAPL2* (At1g61030), that display high similarity to *Wapl* genes characterized in other organisms. The predicted AtWAPL1 (930 amino acids) and AtWAPL2 (840 amino acids) proteins are larger than those from yeast and worm, but shorter than the vertebrate and fly proteins (Figure 1A). AtWAPL1 and AtWAPL2 share 82% amino acid similarity with each other and 30–37% similarity with Wapl proteins from other organisms (Figure S1). Both Arabidopsis proteins contain the conserved Wapl C-terminal domain. The N-terminus of vertebrate Wapl contains FGF motifs that are involved in Pds5 binding [41]. FGF motifs are not present in AtWAPL1, AtWAPL2, or other nonvertebrate Wapl proteins.

### *Arabidopsis WAPL* genes are redundant

**Figure 2:**
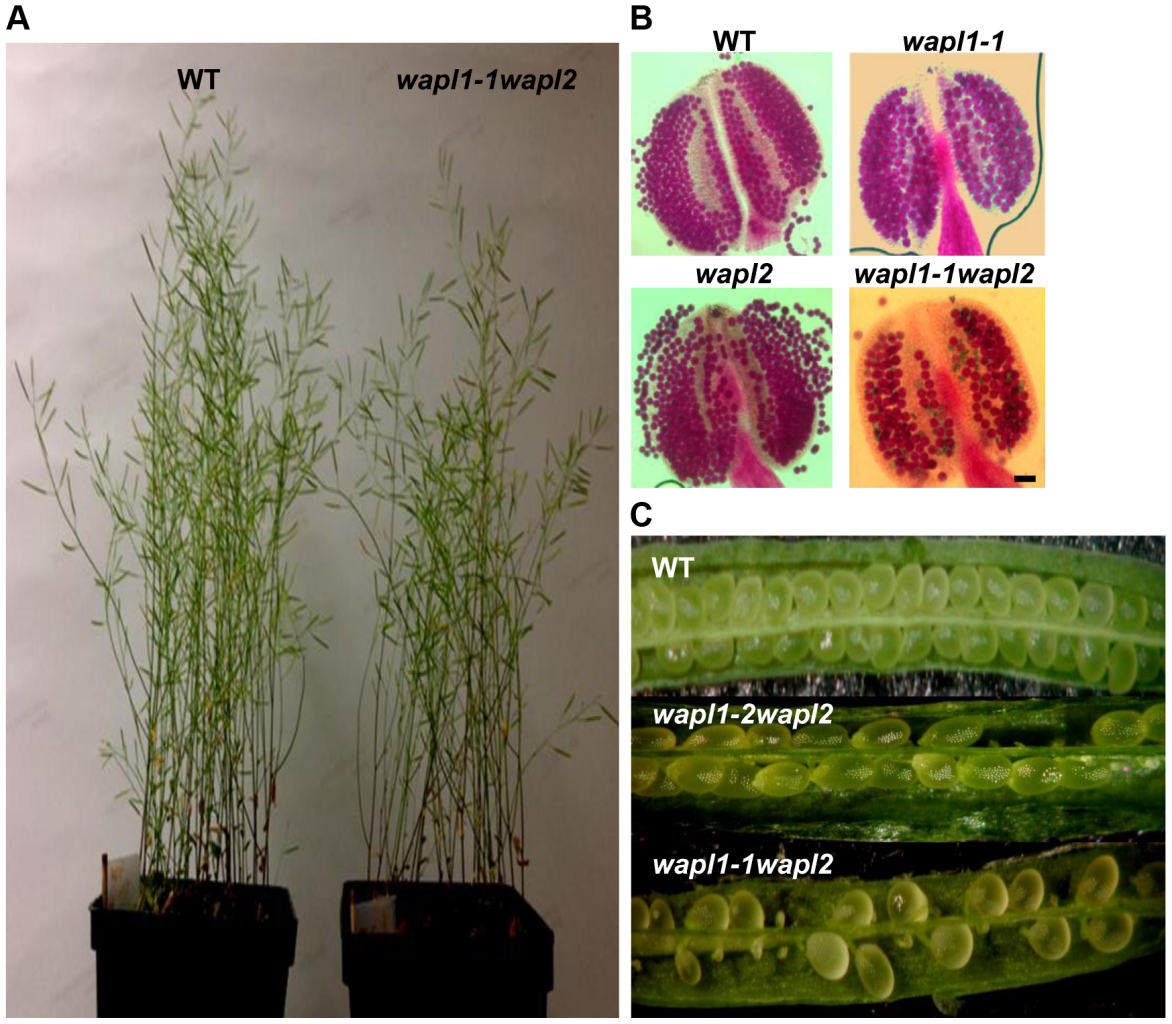
*Atwapl1-1wapl2* plants exhibit reduced fertility. (A) Thirty five day-old wild-type and *Atwapl1-1wapl2* plants. (B) Alexander staining of wild-type, *Atwapl1-1*, *Atwapl2*, and *Atwapl1-1wapl2* pollen. Green pollen is nonviable. Size Bars = 10 µm. (C) Seed setting in siliques of wild type, *Atwapl1-2wapl2* and *Atwapl1-1wapl2* plants.

In order to determine if the two predicted *Arabidopsis WAPL* genes are in fact involved in controlling sister chromatid cohesion, we characterized T-DNA insertion lines that were available in the *Arabidopsis* Stock Center. Two lines were characterized for *AtWAPL1* (*Atwapl1-1* and *Atwapl1-2*, Figure 1B) and one line for *AtWAPL2* (*Atwapl2*, Figure 1B). Plants homozygous for the individual insertion lines displayed normal vegetative growth, development and fertility when compared with wild type plants. The high degree of similarity between *AtWAPL1* and *AtWAPL2* raised the possibility that the two genes share overlapping functions. Therefore, we crossed *Atwapl2* with both *Atwapl1-1* and *Atwapl1-2*. Plants double homozygous for both combinations (*Atwapl1-1wapl2* and *Atwapl1-2wapl2*) were isolated and studied. Plants homozygous for both the *Atwapl1-2* and *Atwapl2* mutations displayed normal vegetative growth and development, but a reduction in fertility. Average seed set/slique in *Atwapl1-2wapl2* plants (43.7±5.1, n = 32) is lower than wild type (53.7±4, n = 42, p<0.0001). Plants containing the *Atwapl1-1wapl2* double mutant combination showed a more pronounced phenotype. Specifically, the plants grew somewhat slower than wild type plants (Figure 2A) and produced shorter siliques, which contained fewer seeds (37.5±6.7, n = 45, p<0.0001) than *Atwapl1-2wapl2* sliques. Further analysis of both double mutant combinations identified similar alterations in reproduction, including aborted pollen and ovules prior to fertilization and embryo defects in approximately 25% of the fertilized seed, with higher numbers of aborted pollen, ovules and seed consistently observed in *Atwapl1-1wapl2* plants (Figure 2B, C).

**Figure 3:**
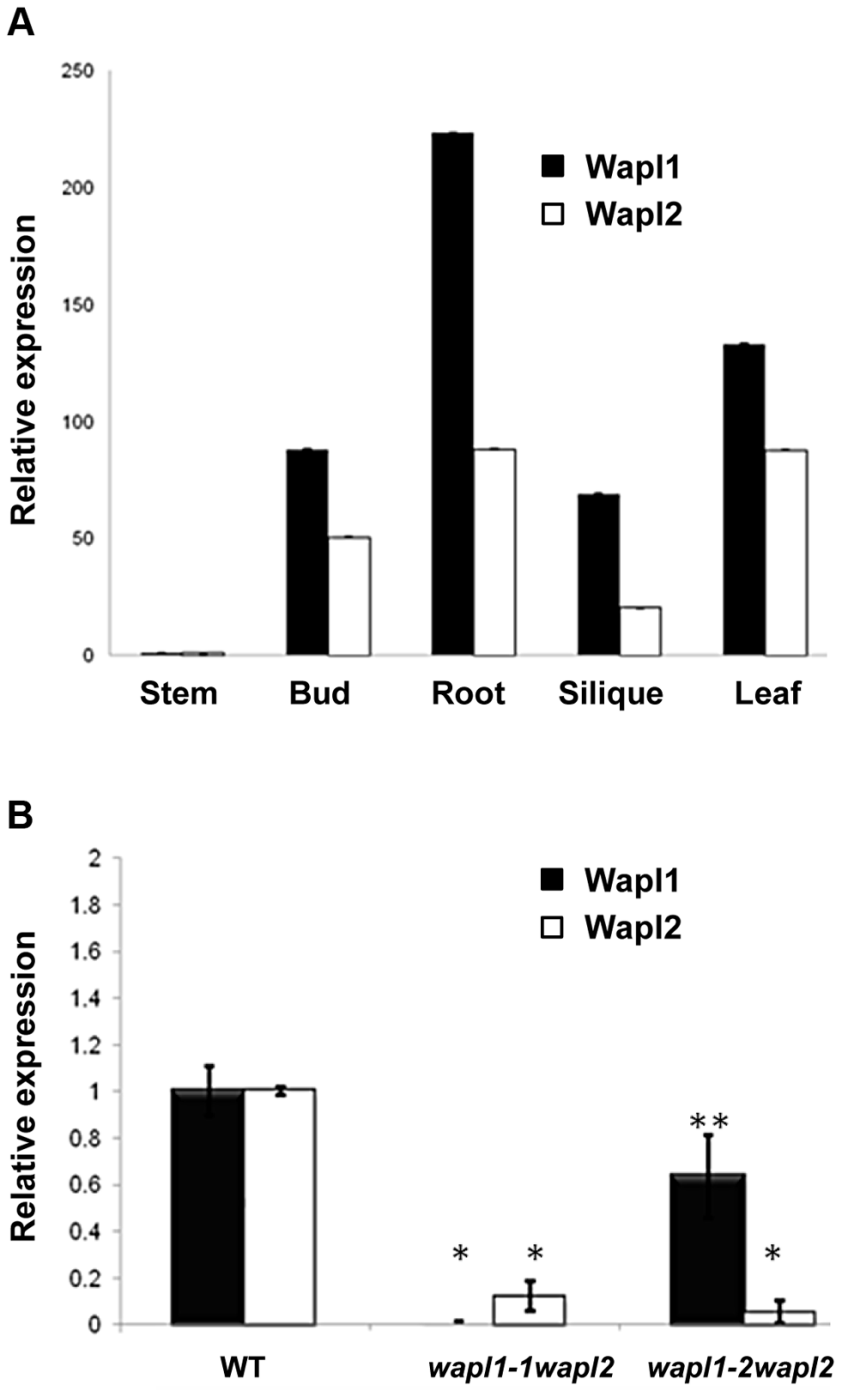
*AtWAPL1* and *AtWAPL2* show similar expression patterns. (A) Relative transcript levels for *AtWAPL1* and *AtWAPL2* in different wild type tissues are shown. (B) *AtWAPL* transcript levels in bud tissue from wild type, *Atwapl1-1wapl2* and *Atwapl1-2wapl2* plants. Results are shown as means ± SD (*n* = 3). Asterisks represent significant differences between mutant and wild type levels (*P<0.0001, **P<0.001; Student's *t*-test).

The *Atwapl1-2* and *Atwapl2* T-DNA insertions are in the first exon and intron, respectively, while the *Atwapl1-1* insert is located in intron 6 (Figure 1B). In order to investigate the differences we observed between the two double mutant combinations and determine if the T-DNA insertions result in complete inactivation of the genes we examined *AtWAPL1* and *AtWAPL2* transcriptional patterns in both wild type and mutant plants. Transcripts for both genes were detected in roots, leaves, buds and sliques of wild type plants; little to no transcript for either gene was detected in stems (Figure 3A). While both genes are active, *AtWAPL1* transcripts were more abundant than those for *AtWAPL2* in all tissues examined, with the highest overall levels observed in roots (Figure 3A). Analysis of *WAPL* transcript levels by qPCR with primers located downstream of the T-DNA inserts in the different double mutant backgrounds indicated that the *Atwapl1-1* mutation effectively results in complete inactivation of the gene. In contrast, RNA corresponding to sequences downstream of the *Atwapl1-2* T-DNA insert were detected at levels approximately 75% of wild type (Figure 3B). The weak phenotype associated with *Atwapl1.2wapl2* plants may be due to the production of reduced levels and/or a partially functional protein from the *Atwapl1.2* allele. Low levels (>10% wild type) of truncated *Atwapl*2 transcripts were also detected downstream of the *Atwapl2* T-DNA insert. This raised the possibility that a small amount of truncated WAPL2 protein may also be produced. The truncated protein would be missing at least the first 136 amino acids of the protein, including a stretch of highly conserved amino acids (Figure S1). Because the *Atwapl1-1wapl2* mutant combination resulted in the most severe phenotype, we confined our more detailed analyses to *Atwapl1-1wapl2* plants.

**Figure 4:**
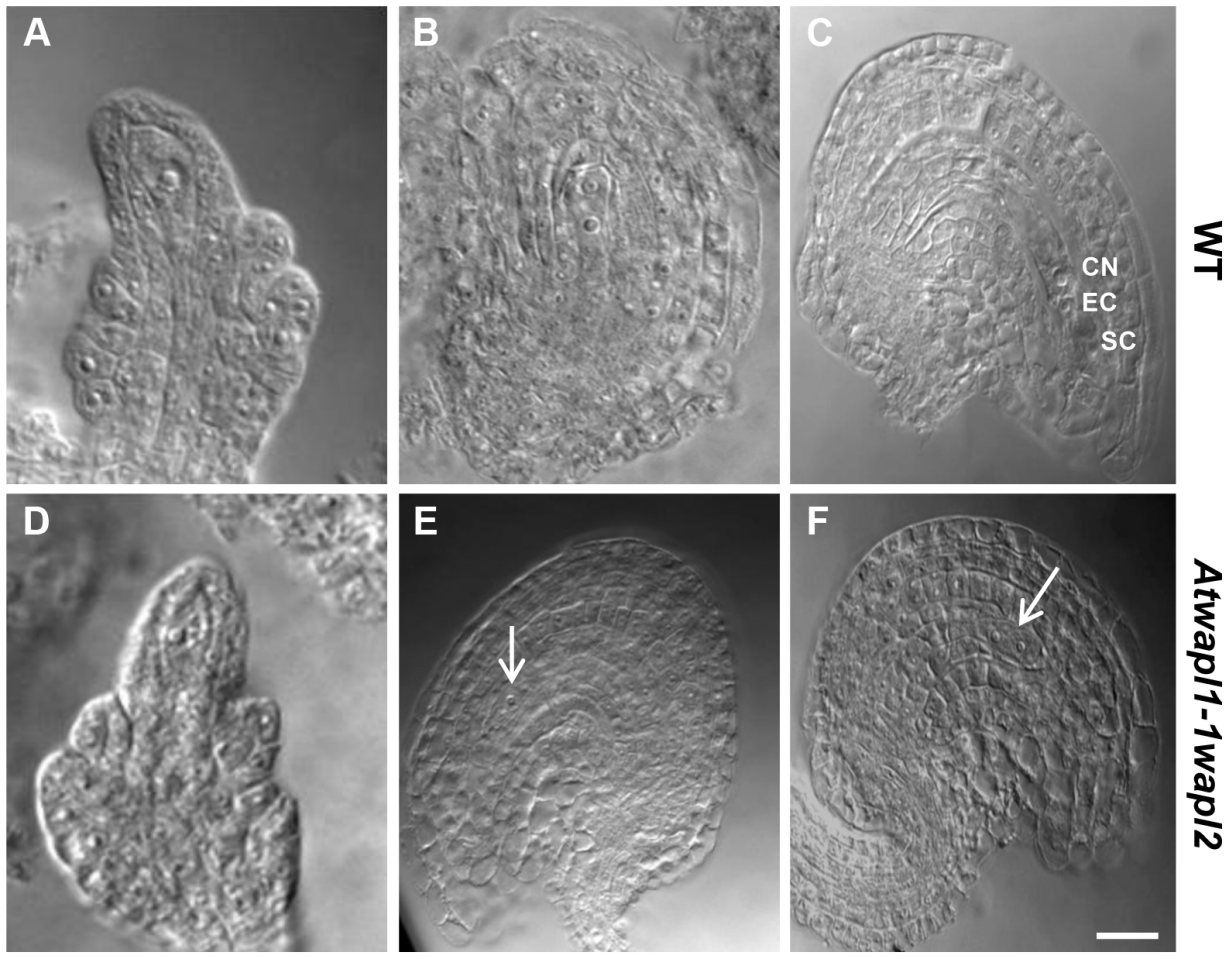
Female gametophyte development is altered in *Atwapl1-1wapl2* plants. Cleared ovules of wild-type (A–C) and *Atwapl1-1wapl2* (D–F) plants are shown at the Megaspore Mother Cell stage (A, D), wild type FG2 (B, E) and wild-type FG7 stages (C, F), CN: Central nucleus, EC: egg cell, SC: synergid cell. Female gametophytes were found to arrest at FG1 (E) and FG2 (F) in *Atwapl1-1wapl2* plants. Images shown for *Atwapl1-1wapl2* represent the most common phenotypes observed. Arrows indicate arrested nuclei. Size bar = 10 µM.

Anthers of *Atwapl1-1wapl2* plants contain less pollen than wild type plants (229±21.3, n = 15 verses 458±23.8, n = 10, p<0.0001) and 28% of the pollen (n = 2752) that is produced is not viable, appearing green and shriveled when analyzed by Alexander stain (Figure 2B). Analysis of seed development in *Atwapl1-1wapl2* plants revealed that 28% of the ovules (n = 1689) abort prior to fertilization, while 23% of the seed (n = 2022) that is produced is shrunken and shriveled. Examination of cleared ovules from developmentally staged siliques of *Atwapl1-1wapl2* plants identified defects beginning after the Megaspore Mother Stage (Figure 4 A, D). Approximately 16% of ovules examined (n = 409) arrest at FG1 with one nucleus (Figure 4E). Approximately eight percent of the ovules arrest at FG2 (Figure 4F). In most instances the arrested nuclei persisted throughout ovule development and were observed in siliques with normal FG7 ovules.

### AtWAPL plays an important role in meiosis

**Figure 5:**
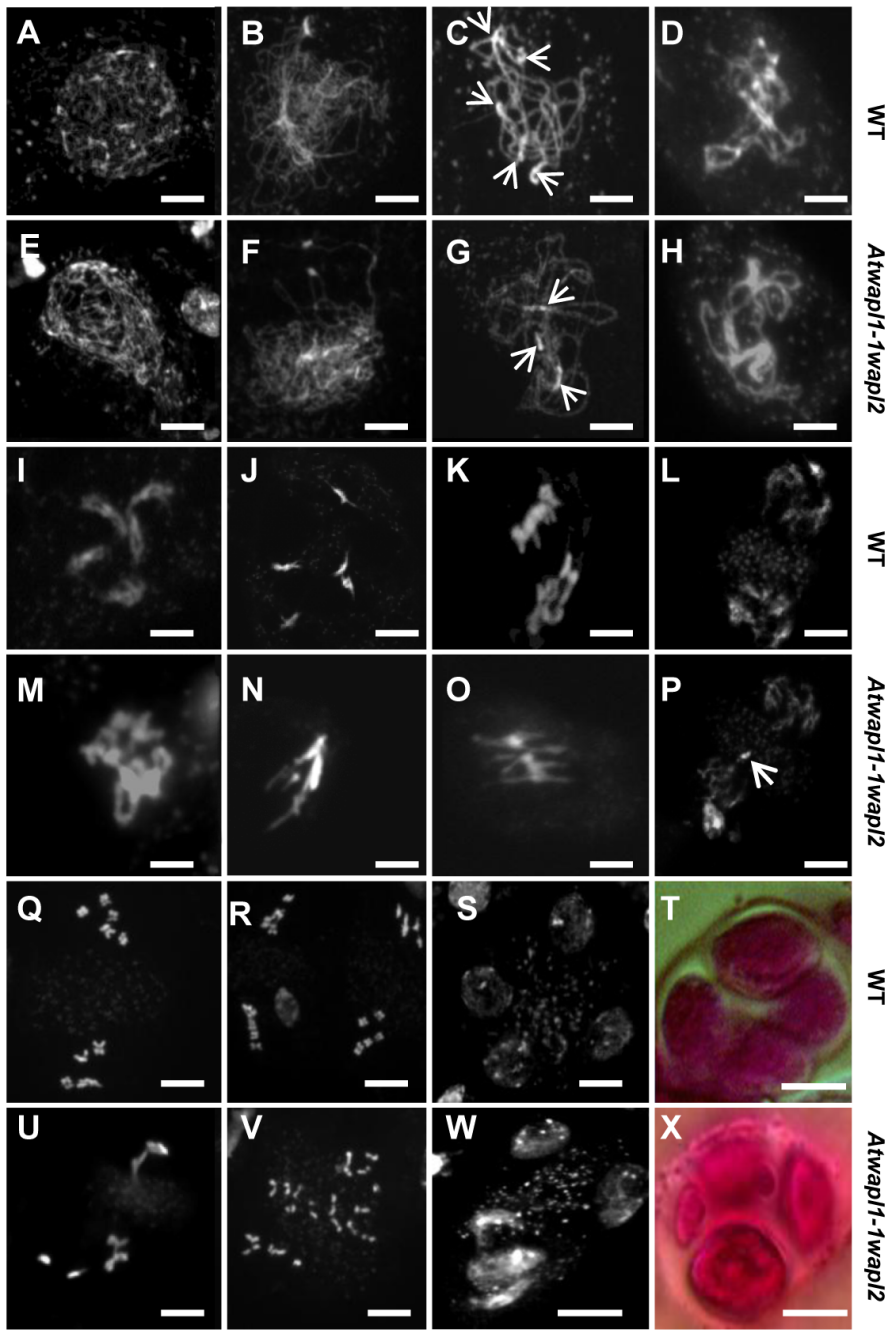
*Atwapl1-1wapl2* plants exhibit defects during male meiosis. DAPI stained chromosomes from male meiocytes of wild type (A–D, I–L, Q–S) and *Atwapl1-1wapl2* plants (E–H, M–P, U–W) are shown at leptotene (A, E), zygotene (B, F), pachytene (C, G), diplotene (D, H), diakinesis (I, M), metaphase I (J, N), anaphase 1 (K, O), telophase I (L, P), metaphase II (Q, U), telophase II (R, V) and tetrad stage (S, W). Alexander stained tetrads/polyads are shown in (T, X). Images shown for *Atwapl1-1wapl2* represent the most common phenotypes observed at each stage. Arrows in C & G denote chromocenters. Arrow in P denotes a lagging chromosome. Size bar = 5 µm.

The presence of aborted ovules and reduced numbers of pollen in *Atwapl1-1wapl2* plants suggested that AtWAPL plays an important role in meiosis. To investigate this possibility further we analyzed DAPI (4′, 6-diamidino-2-phenylindole) stained meiotic chromosomes in *Atwapl1-1wapl2* plants. Early stages of meiosis appeared relatively normal in the mutant. As observed in wild type meiocytes, chromosomes began to condense as fine thin threads during leptotene (Figure 5A, E) and homologous chromosome co-alignment and pairing occurred during early to mid zygotene (Figure 5B, F). In wild type meiocytes homologous chromosomes are fully synapsed by the beginning of pachytene (Figure 5C). While most late zygotene/pachytene stage meiocytes exhibited normal synapsis, in 15% of the *Atwapl1-1wapl2* pachytene meiocytes (n = 135) the chromosomes co-aligned but did not synapse completely (Figure 5G). In addition, four to six brightly stained chromocenters are typically observed in wild type meiocytes, while in the mutant we observed three or fewer heterochromatin regions in 60% of the *Atwapl1-1wapl2* pachytene cells, suggesting that abnormal association of heterochromatic regions may occur in the mutant.

Desynapsis occurs during diplotene (Figure 5D) with five bivalents appearing at diakinesis in wild type meiocytes (Figure 5I). The five bivalents align at the equatorial plane at metaphase I (Figure 5J). Segregation of homologous chromosomes and then sister chromatids at anaphase I and anaphase II, respectively, results in the presence of four sets of five individual chromosomes at the cell poles by telophase II (Figures 5K, L and Q, R). Early diplotene appeared relatively normal in the mutant (Figure 5H). However, alterations were observed by diakinesis in essentially all cells. Specifically meiocytes were observed in which the chromosomes condensed into either one or two large intertwined masses of chromatin (Figure 5M, n = 25). The chromosomes continued to appear primarily as one intertwined mass as they further condensed and moved to the cell equator; five normal appearing individual bivalents were never observed (Figure 5N, n = 23). While some (<20%) normal cells were observed at the metaphase I-anaphase I transition, most cells contained stretched chromosomes that did not separate properly (Figure 5O, n = 57). Chromosome bridges and lagging chromosomes were observed by late anaphase I and telophase I (Figure 5P, n = 31), respectively in the majority of meiocytes. In most cells (68%, n = 31) “sticky” chromosome masses were observed at one or both poles at metaphase II (Figure 5U); however in approximately 30% of the meiocytes individual chromosomes appeared to align normally. Twenty or more chromosomes/chromosome fragments were typically observed scattered around most (62%, n = 26) anaphase II and telophase II cells (Figure 5V). Ultimately, a mixture of polyads (6%), tetrads (26%) containing a mixture of shrunken and mis-shaped microspores with varying amounts of DNA (Figure 5W, X), and relatively normal appearing tetrads were observed (n = 506).

### WAPL helps prevent abnormal centromere association during prophase I

**Figure 6:**
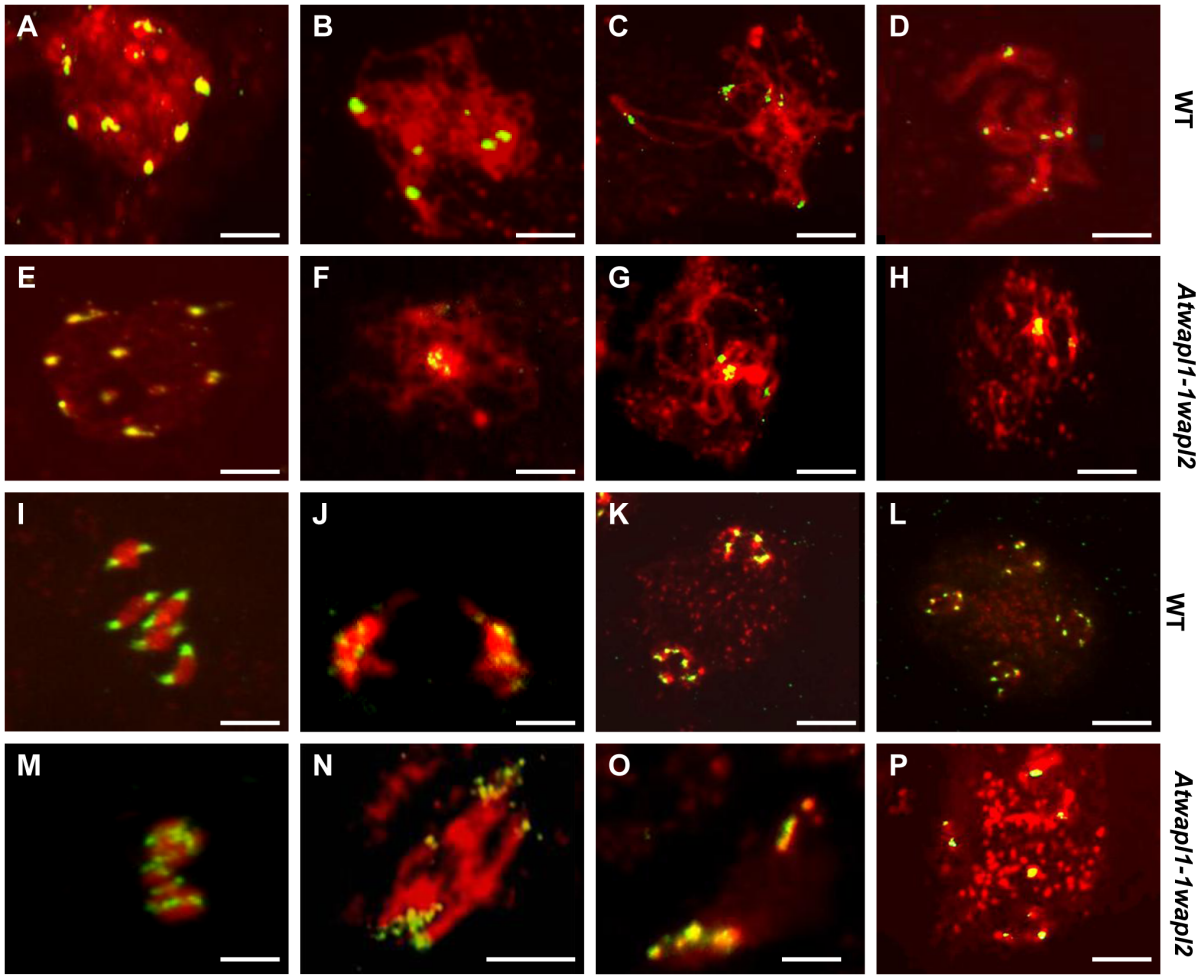
*Atwapl1-1wapl2* meiocytes exhibit nonspecific association of centromeres. FISH was conducted using a 180(A–D, I–L) and *Atwapl1-1wapl2* (E–H, M–P) plants. DAPI-stained chromosomes are shown in red, centromere FISH signals in green. Ten signals are observed at interphase I cells of both lines (A, E). Five signals are typically observed during zygotene (B), pachytene (C), and diplotene (D) in wild type meiocytes. Clusters of centromere signals are typically observed in *Atwapl1-1wapl2* meiocytes during prophase I (F, G, H). In wild type five pairs of chromosomes are observed at metaphase I (I) that separate into two groups of five signals at anaphase I (J); two groups of five pairs of signals are observed at metaphase II (K) followed four groups of five signals at telophase II (L). Ten to twenty signals that show aberrant segregation are observed from anaphase I onward in *Atwapl1-1wapl2* meiocytes (M–P). Images shown for *Atwapl1-1wapl2* represent the most common phenotypes observed at each stage. Size bar = 10 µm.

One of the earliest defects observed in the meiocytes of *Atwapl1-1wapl2* plants is a reduced number of heterochromatin regions, suggesting that AtWAPL is important early in prophase I, possibly in controlling heterochromatin structure. In order to investigate this possibility, fluorescence *in situ* hybridization (FISH) experiments were conducted using a 180 bp repetitive centromere fragment as a probe on meiocytes of wild type and *Atwapl1-1wapl2* plants. Eight to ten centromere signals were observed in meiocytes during leptotene in both wild type (mean = 9.2±0.71, n = 26) and *Atwapl1-1wapl2* (mean = 9.0±1.2, n = 29) plants (Figure 6 A, E). Four to six signals were normally observed in wild type meiocytes (mean = 5.4±0.5, n = 25) during zygotene as homologous chromosomes pair (Figure 6B). Alterations were first observed at zygotene when approximately 50% of the *Atwapl1-1wapl2* meiocytes observed (n = 30) were found to contain clusters of condensed signals (Figure 6F). At pachytene wild type and *Atwapl1-1wapl2* meiocytes contained on average 4.8±0.35 (n = 8) and 3.04±1.3 (n = 84) centromere signals, respectively with 50% of *Atwapl1-1wapl2* meiocytes showing one or two clusters of signals (Figure 6 C, G). Five pairs of centromere signals corresponding to the five bivalents are visible at diakinesis and early metaphase I in wild type meiocytes, followed by ten signals during anaphase I/telophase I and 20 during meiosis II (Figure 6D, I–L, n = 48). In contrast, centromere signals continued to cluster together at late diplotene and diakinesis (Figure 6H) in 60% of the *Atwapl1-1wapl2* meiocytes examined (n = 24). Individual centromere signals could however be observed within the condensed chromatin at metaphase I (n = 15) (Figure 6M). While some normal anaphase I cells were observed, more than ten centromere signals were observed beginning at anaphase I in 65% of the *Atwapl1-1wapl2* meiocytes observed (n = 27), suggesting that either centromere cohesion is lost prematurely or never properly formed in these cells. Approximately 35% of the cells proceed normally through the remainder of meiosis. However, in most cells centromere signals of varying intensities were observed that associated with mis-segregated chromosomes and chromosome fragments at telophase I (Figure 6N) and chromosomes scattered around the cells during meiosis II (Figure 6O, P, n = 24).

**Figure 7:**
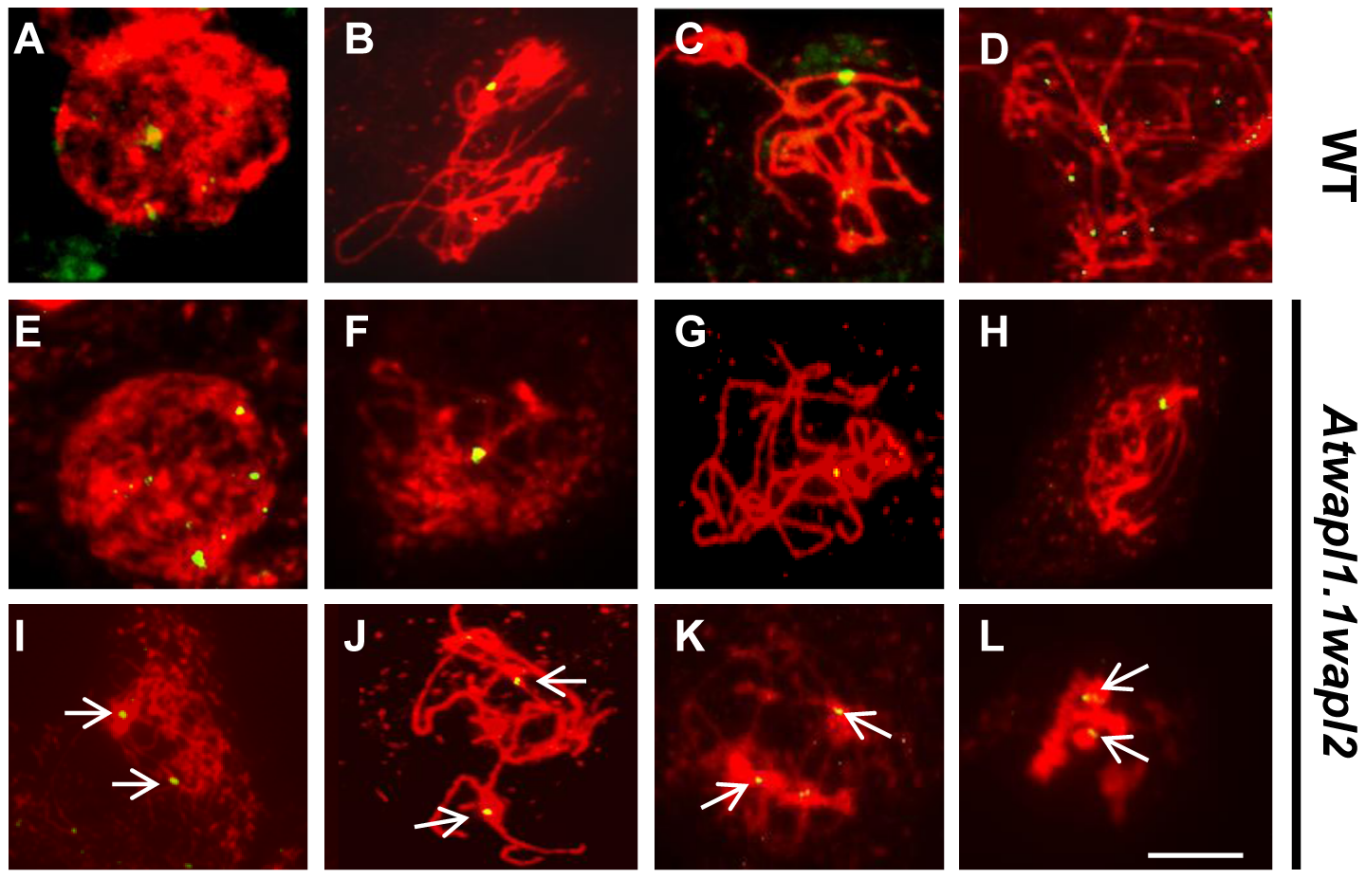
*Atwapl1-1wapl2* meiocytes exhibit alterations in homologous chromosome pairing. FISH was conducted on male meiocytes from wild type (A–D) and *Atwapl1-1wapl2* (E–L) plants using a telomere repeat probe that binds to a region proximal to the centromere of chromosome 1. Two signals are observed at early leptotene (A, E, I). One signal reflecting synapsed chromosomes is observed at late zygotene and pachytene in wild type and some *Atwapl1-1wapl2* meiocytes (B, C F, G), while two signals are observed in others (J, K). Two closely spaced signals are typically observed at diplotene in wild type and many *Atwapl1-1wapl2* meiocytes (D, H) with two widely separated signals in others (L). Images shown for *Atwapl1-1wapl2* represent the most common phenotypes observed at each stage. Size bar = 10 µm.

Results from our chromosome spreading suggested that defects in homologous chromosome pairing and synapsis may also exist in the mutant. To investigate this possibility further we performed FISH using a telomere-derived fragment that also strongly labels a region proximal to the centromere of chromosome 1 [42]. Two strong chromosome 1 signals with weaker telomere signals were observed during leptotene in both wild type (n = 17) and *Atwapl1-1wapl2* (n = 24) meiocytes (Figure 7A, E). One strong signal was observed in wild-type meiocytes starting at zygotene and extending through diplotene (mean = 1.02±0.17, n = 36) (Figure 7B–D). Cells with either one or two chromosome 1 signals were observed during these stages in *Atwapl1-1wapl2* plants. While most cells resembled wild type meiocytes and contained one signal (mean = 1.19±0.40) during zygotene, pachytene and diplotene (Figure 7F–H), approximately 20% of the nuclei observed (n = 139) contained two widely spaced chromosome 1 signals throughout prophase (Figure 7I–L). Therefore, a small but significant fraction of meiocytes do not undergo normal pairing and synapsis.

Meiotic prophase was investigated further by analyzing the distribution of ASY1 and ZYP1. ASY1 is a meiosis-specific protein that is intimately associated with chromosome axes during prophase I. Differences were not observed in ASY1 labeling between wild type and *Atwapl1-1wapl2* meiocytes (Figure S2). In both wild type and *Atwapl1-1wapl2* meiocytes ASY1 appears as diffuse foci during G2, forming thin threads that co-localize with the developing univalent axes during leptotene. It is associated with the axes of the synapsed chromosomes during pachytene and disappears from chromosomes at diplotene. Subtle alterations were however observed in ZYP1 distribution in approximately 25% of the meiocytes. ZYP1, an axial element protein, appears at zygotene as foci. ZYP1 signals extend during pachytene producing a continuous signal between the synapsed homologous chromosomes [43]. The majority (77%) of *Atwapl1-1wapl2* pachytene meiocytes examined (n = 30) resembled wild type and exhibited continuous ZYP1 signals. However, 23% of the meiocytes exhibited more diffuse ZYP1 labeling patterns and contained pachytene chromosomes that exhibited discontinuous and/or unpaired ZYP1 signals (Figure S3). Therefore, while ASY1 and ZYP1 appear to load normally on *Atwapl1-1wapl2* meiotic chromosomes, some meiocytes do not undergo complete synapsis.

### WAPL determines the timely release of meiotic cohesion

**Figure 8:**
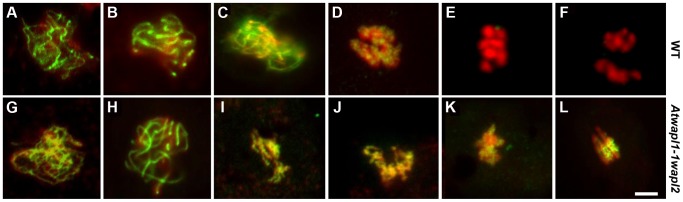
Cohesin release is delayed in *Atwapl1-1wapl2* meiocytes. Meiotic spreads of wild type (A–F) and *Atwapl1-1wapl2* (G–L) plants were stained with anti-SYN1 antibody (green) and propidium iodide (red). Meiocytes in wild-type and *Atwapl1-1wapl2* plants exhibited similar SYN1 staining at zygotene (A, G) and pachytene (B, H). SYN1 is removed from the arms of wild type meiocytes during diplotene (C) and diakinesis (D) and is not detectable during metaphase I and anaphase I (E, F). Strong SYN1 signal is observed on the chromosomes of *Atwapl1-1wapl2* meiocytes during diplotene, diakinesis, metaphase and anaphase (I–L). Images shown for *Atwapl1-1wapl2* represent the most common phenotypes observed at each stage. Size bar = 5 µm.

The observed alterations in chromosome structure and the “sticky” nature of meiotic chromosomes suggested that *Atwapl1-1wapl2* plants may be defective in the release of cohesin during prophase. In order to investigate this possibility, we performed immunolocalization experiments on *Atwapl1-1wapl2* and wild type meiocytes with antibodies to SYN1, the Arabidopsis homolog of REC8 [44]. Cohesin labeling appeared normal in *Atwapl1-1wapl2* plants during early stages of prophase I. At interphase SYN1 exhibited diffuse nuclear labeling with the signal decorating the developing chromosomal axes beginning at early leptotene and extending into zygotene. During late zygotene and pachytene the protein lined the chromosomes (Figure 8A, B, G, H). A large amount of SYN1 is released from wild type meiotic chromosomes during diplotene (Figure 8C, n = 9) and diakinesis (Figure 8D, n = 7) as the chromosomes condense. By prometaphase I SYN1 is barely detectable on wild type chromosomes (Figure 8E, n = 14). In contrast, strong SYN1 labeling was consistently observed from diplotene into anaphase I in the mutant (Figure 8I–L). SYN1 was observed on “sticky” metaphase I chromosomes (Figure 8K, n = 5) and stretched bivalents during anaphase I (Figure 8L, n = 10). While 20% of metaphase II meiocytes (n = 25) showed faint SYN1 signals, the majority of meiocytes did not, suggesting the protein is removed during telophase I and interphase II.

### WAPL is important for proper spindle attachment and assembly during meiosis

**Figure 9:**
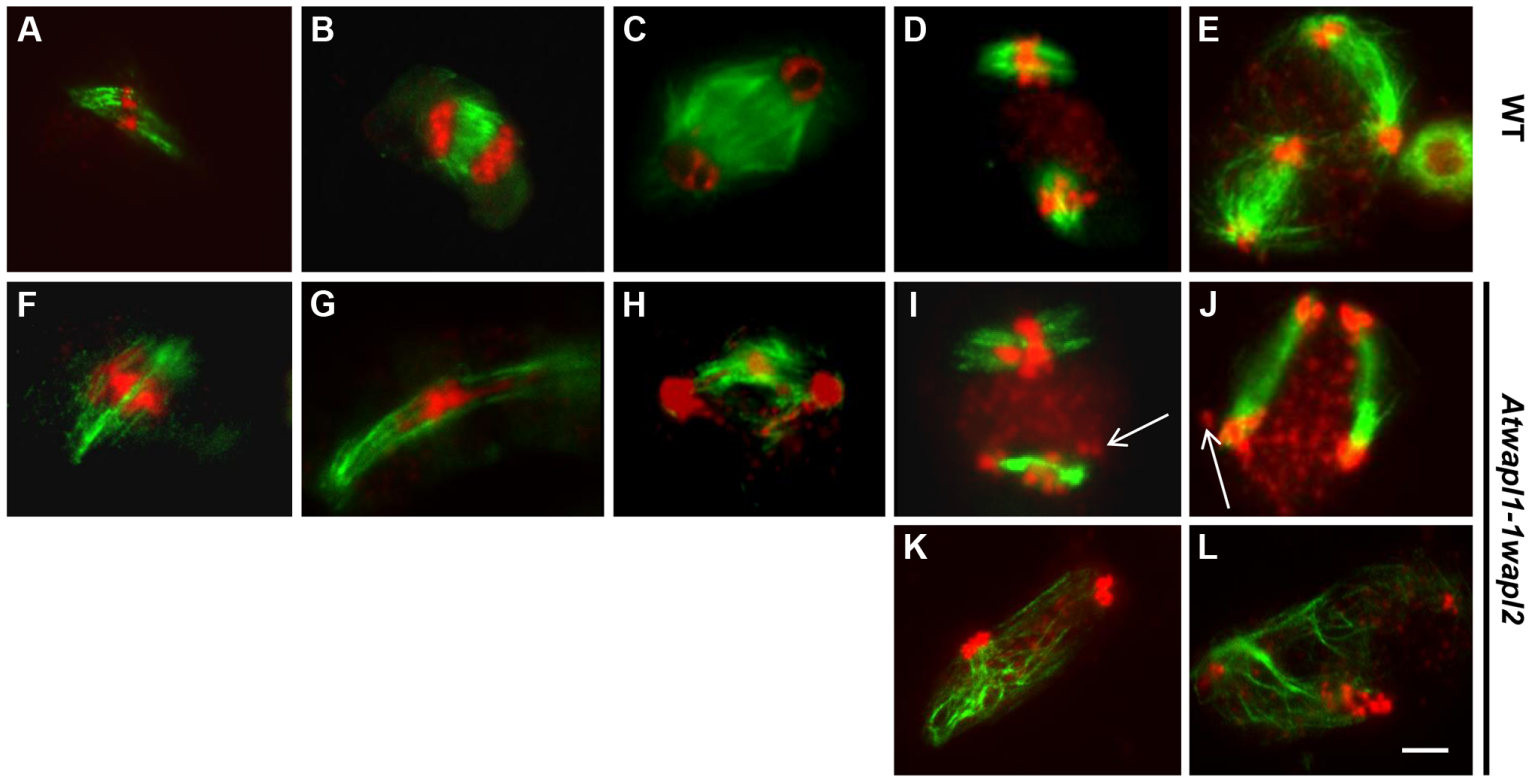
Meiotic spindle assembly and structure is altered in *wapl* plants. Spindles of male meiocytes from wild type and (A–E) and *Atwapl1-1wapl2* (F–L) plants were stained with anti-β tubulin antibody (green) and DNA was counterstained with propidium iodide (red). Alterations were observed in *Atwapl1-1wapl2* male meiocytes throughout meiosis, including metaphase I (F), anaphase I (G), telophase I (H), metaphase II (I, K), and telophase II (J, L). Images shown in F–H represent those most commonly observed during meiosis I in *Atwapl1-1wapl2* male meiocytes, while those in I, K and J, L represent the two most common classes of defects observed in metaphase II and telophase II, respectively. Size bar = 10 µm.

As part of our studies to better define meiotic stages in the mutant and further characterize chromosome behavior, we performed immunolocalization studies using β-tubulin antibody on wild type and *Atwapl1-1wapl2* meiocytes. No significant differences in β-tubulin labeling were observed between wild type and mutant plants during interphase and prophase I. Wild type spindles exhibit a bipolar configuration during metaphase I and anaphase I (Figure 9A, B), with radial spindles forming between the two groups of chromosomes at telophase I (Figure 9C). Two bipolar spindles, which are perpendicular to each other, are then observed during metaphase II and anaphase II (Figure 9D, E), with radial microtubules again forming between the four separated nuclei during telophase II.

While normal bipolar spindles were formed during metaphase I and metaphase II in approximately 35% of *Atwapl1-1wapl2* meiocytes, the majority of cells showed abnormal spindle configurations. For example, cells in which spindle microtubules passed over the chromosomes were observed (Figure 9F, n = 20). During anaphase I spindles were commonly stretched and not well defined (Figure 9G, n = 31), with alterations being observed in the radial spindles during telophase I (Figure 9H, n = 14) and interphase II. Two types of alterations were commonly observed during meiosis II. Approximately 30% of metaphase II cells contained parallel spindles (Figure 9I, J, n = 12), while another 30% of the cells lacked metaphase II spindles altogether and instead contained random microtubule networks (Figure 9K, L, n = 13). A large number of additional alterations, including cells lacking metaphase I spindles, stretched metaphase II spindles, and cells with four bipolar or parallel spindles were observed at lower frequencies (Figure S4).

### WAPL is required for early embryonic patterning

**Figure 10:**
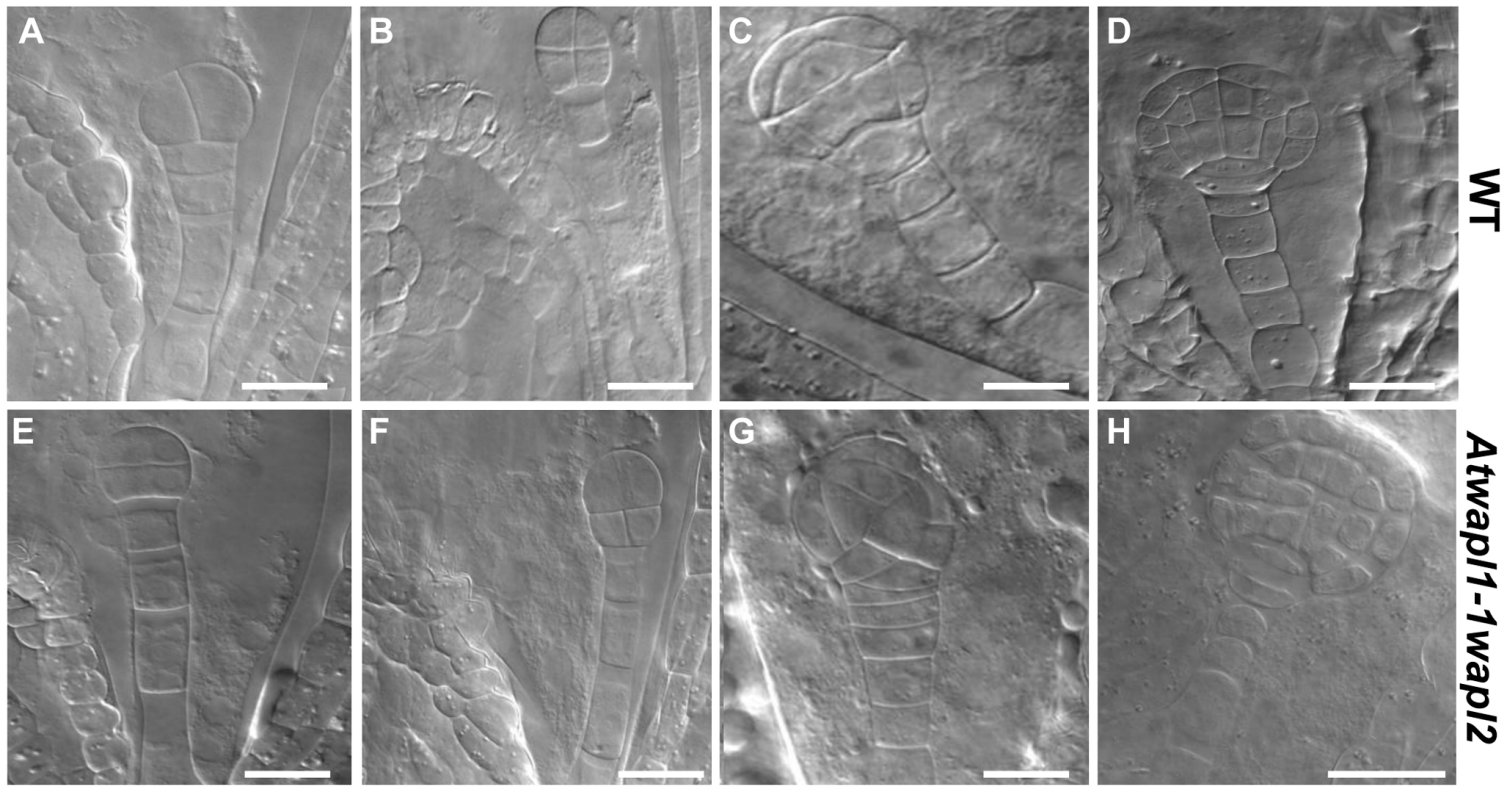
Embryonic patterning is defective in the seeds of *Atwapl1-1wapl2* plants. Fertilized ovules of wild type (A–D) and *Atwapl1-1wapl2* (E–H) plants were cleared in Hoyers solution and viewed using DIC microscopy. Abnormal division planes were observed early in development, including in two (E) and four celled embryos (F). Asynchronous/abnormal cell division and growth was observed (F, G) with defects becoming more pronounced at the dermatogen (G) and globular stages (H). Images shown for *Atwapl1-1wapl2* represent the most common abnormal phenotypes observed at each stage. Size bar = 10 µm.

The siliques of *Atwapl1-1wapl2* plants contain approximately 25% aborted seed (n = 2022), suggesting defects in embryo and/or endosperm development. In order to investigate this possibility we examined cleared seeds in siliques of self-fertilized *Atwapl1-1wapl2* plants and found that 23% of the seed contained abnormal embryos (n = 31 siliques). Alterations in embryo development were observed as early as the two cell stage when instead of the typical vertical division of the apical cell, 9% the mutant embryos (n = 61) performed a horizontal division (Figure 10A, E). Alterations in the suspensor were also observed early in development in approximately 5% of the seeds (n = 39). Suspensors with either two cells instead of a file of four cells and suspensors with abnormal shapes were observed (Figure S5A). A common alteration at later stages involved embryos exhibiting altered division planes and shapes (Figure 10G, n = 10). Another common defect involved either abnormal or uncontrolled division in cells destined to become the suspensor hypophysis (Figure 10G, n = 14). In early cotyledon stage siliques, both normal-appearing and abnormal embryos that were either arrested or delayed were observed at several developmental stages, including: dermatogen, globular and early heart stages (Figure 8H, S5). Shrunken seeds with no trace of an embryo were also observed.

Alterations in embryo development could result from the *wapl* mutations directly affecting cellular division in the embryo or from fertilization events involving abnormal gametes. Results from an analysis of embryo development in reciprocal crossing experiments and the analysis of *wapl1-1wapl2^+/−^* and *wapl2wapl1-1^+/−^* plants suggest that the embryo defects may result from multiple factors. When *wapl1-1wapl2* was used as the female in crosses with wild type pollen 2.9% of the seed was defective (n = 105) with no sign of embryo development, similar to wild type crossing experiments (2%, n = 125). In contrast, when wild type females were crossed with *wapl1-1wapl2* pollen, 12.3% of the embryos (n = 173) were defective, exhibiting altered divisional planes and defective suspensors. An additional 2.8% of the seed showed no sign of embryo development, similar to wild type. Embryo defects were also observed in self fertilized *wapl1-1wapl2^+/−^* (8.6%, n = 214) and *wapl2wapl1-1^+/−^* (7.1%, n = 197) plants. These results clearly show that alterations associated with *wapl1-1wapl2* pollen are sufficient to produce embryos with altered divisional planes and defective suspensors. However, the frequency of defective embryos is doubled when both the sperm and egg carry the *wapl* mutations, suggesting a synergistic effect. Further experiments are required to better define the underlying basis for the defects.

### Mitotic cells show chromosome segregation defects, but normal cohesin release

**Figure 11:**
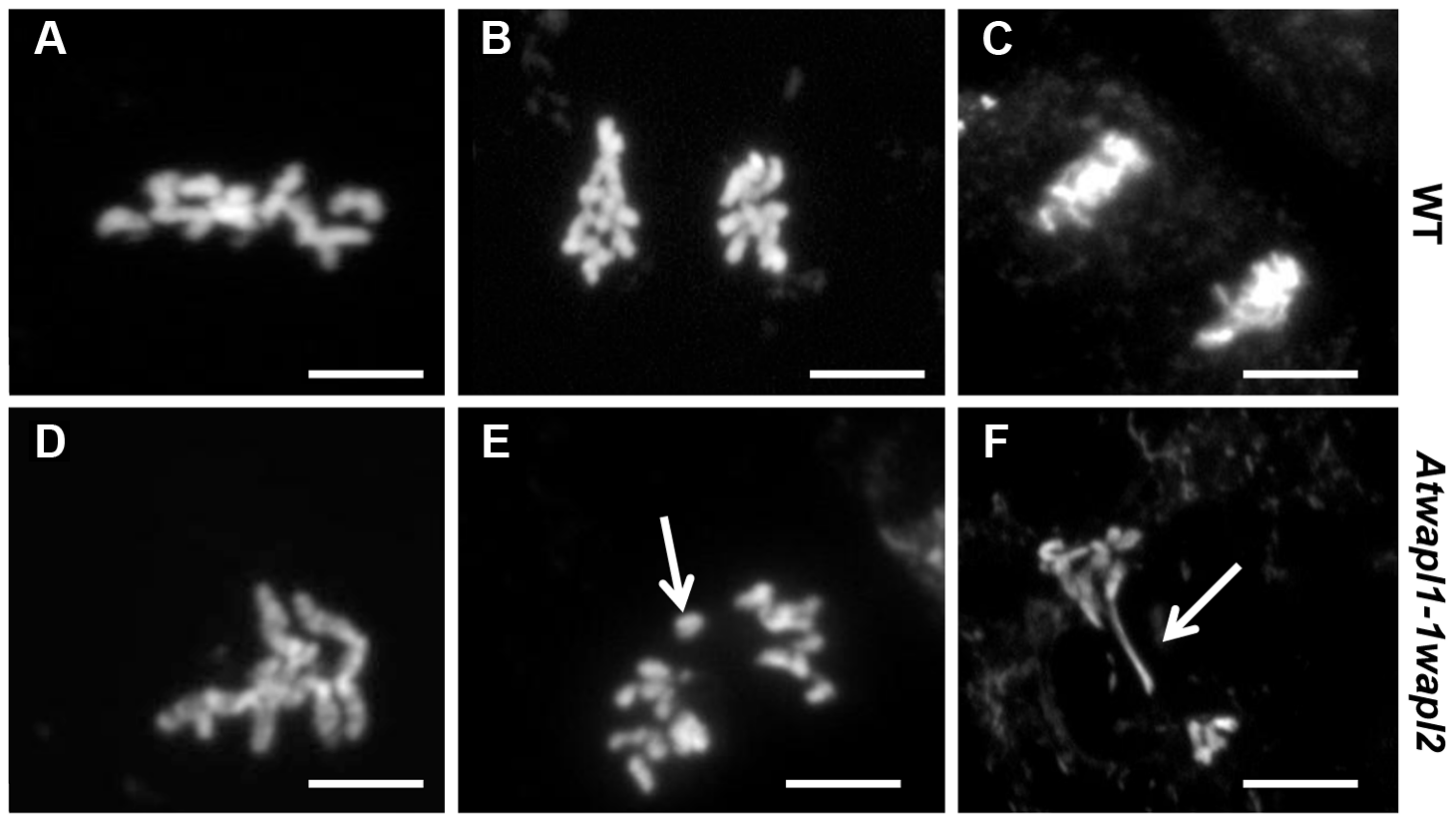
*Atwapl1-1wapl2* plants show defects in mitosis. Root tips of wild type (A–C) and *Atwapl1-1wapl2* plants (D–F) were squashed and stained with DAPI. In wild type root tips the replicated chromosomes condense and align on the metaphase plate (A) followed by the even segregation of ten chromosomes to each pole during anaphase (B) and telophase (C). Most *Atwapl1-1wapl2* root tip cells appeared normal; however 20% of the cells contained metaphase chromosomes that appeared sticky (D). Uneven segregation of chromosomes, chromosome bridges, stretched chromosomes and chromosome fragments were subsequently observed at anaphase and telophase (E, F). Arrows denote a lagging chromosome and chromosome bridge in E and F, respectively. Size Bar = 10 µm.

The fact that *Atwapl1-1wapl2* plants grow and develop normally, albeit slightly slower than wild type suggested that WAPL does not play a major role in nuclear division in somatic cells. In order to determine if *WAPL* mutations have an effect on mitotic cells we examined root tips of *Atwapl1-1wapl2* plants. The majority of mitotic figures observed in the root tips of *Atwapl1-1wapl2* plants (n = 120) appeared normal, with ten pairs of chromosomes condensing at the metaphase plate and then segregating at anaphase/telophase (Figure 11A–C). Altered mitotic figures were however observed in approximately 20% of the cells, with most of the alterations resembling those observed in meiotic cells. The most common alterations were the presence of “sticky chromosomes” at metaphase (Figure 11A, D) that failed to segregate properly at anaphase (Figure 11B, E) resulting in chromosome bridges, lagging chromosomes and possibly chromosome fragments at telophase (Figure 11C, F).

**Figure 12:**
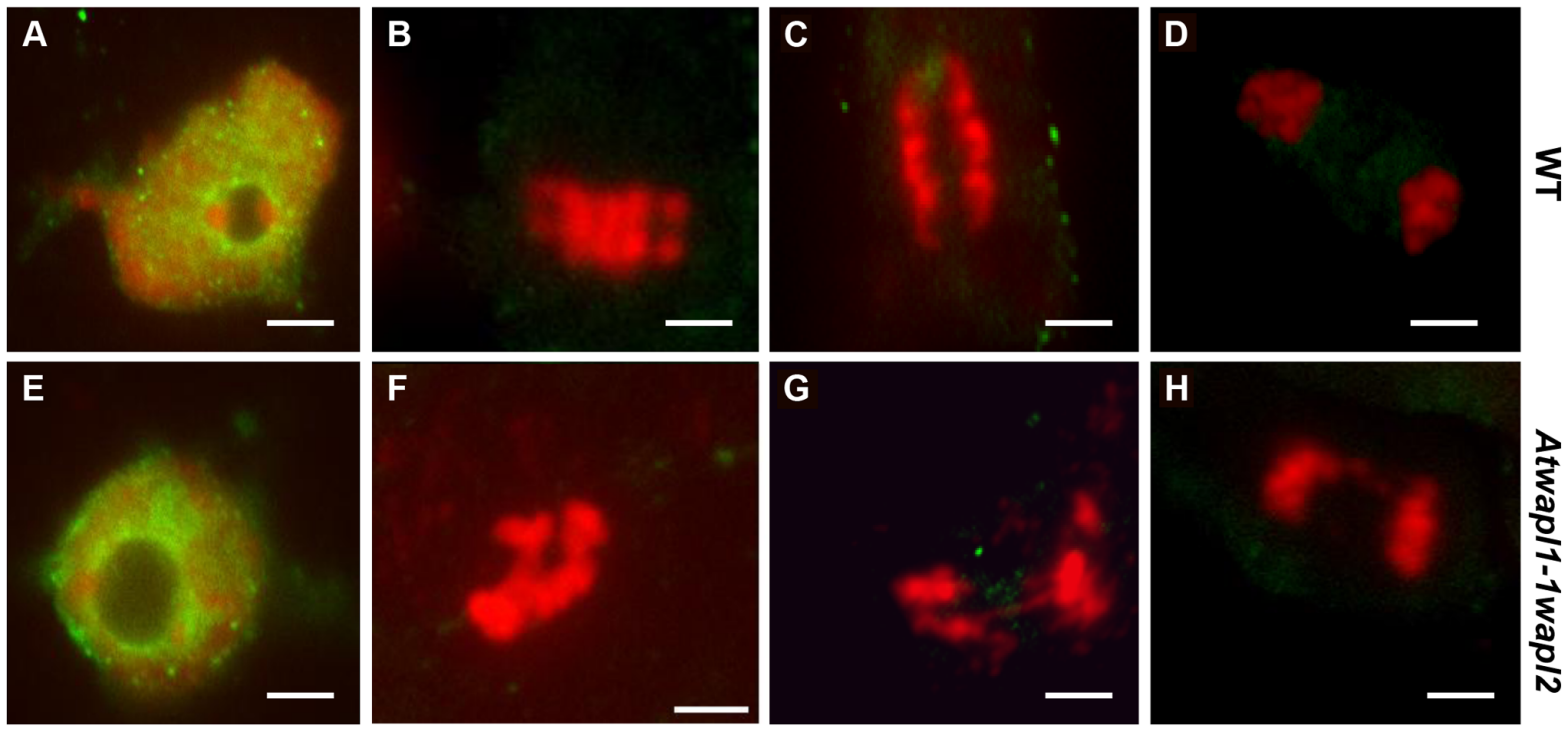
Cohesin is released normally in *Atwapl1-1wapl2* root tip cells. Mitotic spreads of wild type (A–D) and *Atwapl1-1wapl2* (E–H) root tips were prepared and stained with anti-SMC3 antibody (green) and propidium iodide (red). Wild type and *Atwapl1-1wapl2* plants exhibit similar staining patterns during interphase (A, E), metaphase (B, F), anaphase (C, G) and telophase (D, H). Size bar = 5 µm.

Immunolocalization using antibody to SMC3 [35] was performed on root tips of *Atwapl1-1wapl2* plants to determine if cohesin is released normally during mitotic prophase. SMC3 displayed a diffuse labeling pattern during interphase in both wild type and *Atwapl1-1wapl2* plants (Figure 12A, E). The chromosome bound SMC3 signal gradually decreased during prophase and was absent from the chromosomes by metaphase in both wild type and *Atwapl1-1wapl2* plants (Figure 12B, F). Although weak SMC3 signals were sometimes observed, chromosome bound SMC3 signal was never observed (n = 20) during anaphase and telophase (Figure 12C, D, G, H), even on “sticky” metaphase chromosomes or chromosome bridges during anaphase and telophase (Figure 12F–H). Therefore, mitotic cohesin complexes appear to be removed normally during mitosis. However, we can not rule out the possibility that small amounts of cohesin remain on the chromosomes leading to the mitotic alterations we observe.

### *WAPL* mutations rescue *Atctf7*-induced lethality

**Figure 13:**
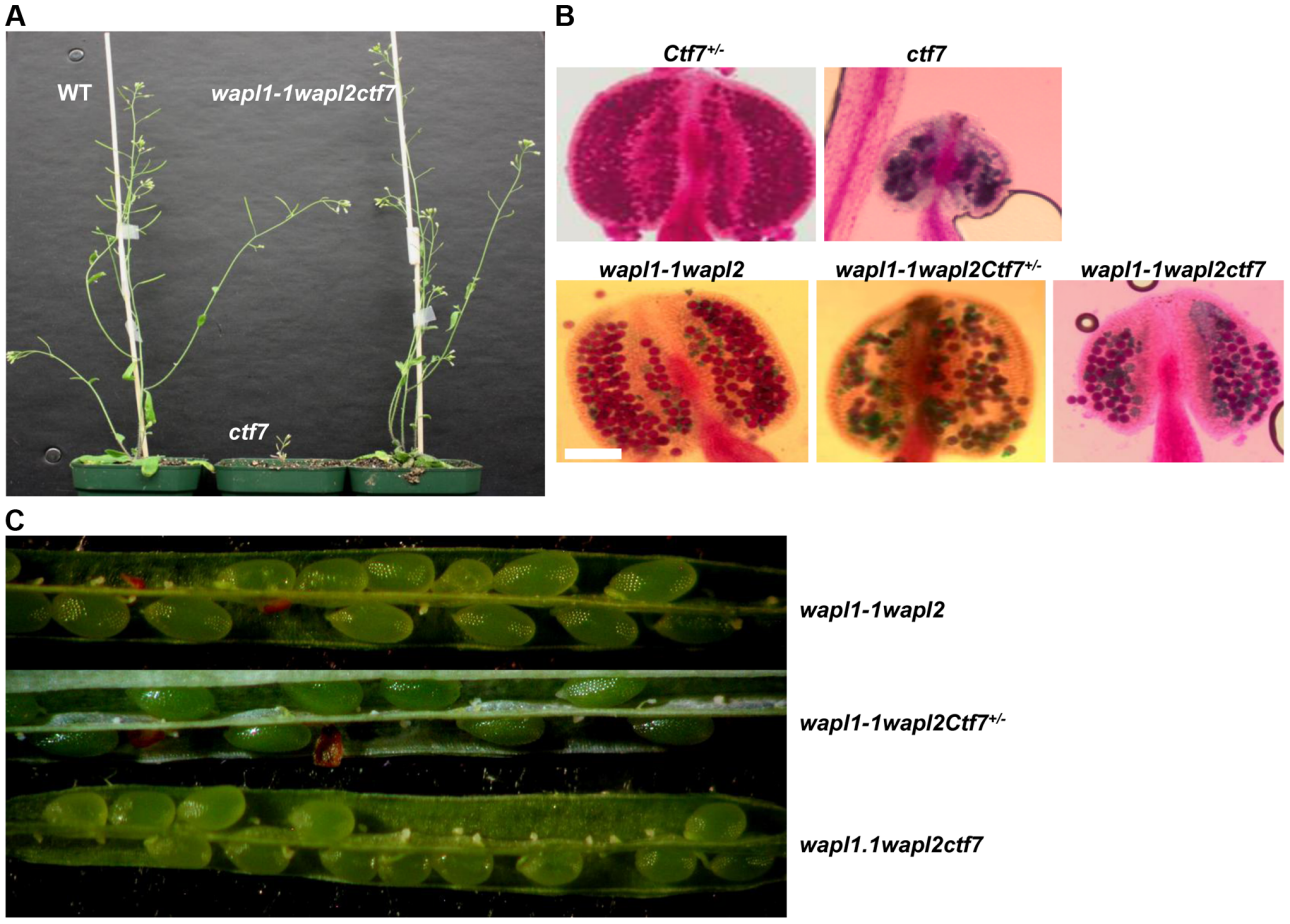
Inactivation of *AtWAPL* rescues *Atctf7* mutants. (A) Thirty day-old wild-type (left), *Atctf7* homozygous (middle) and *Atwapl1-1wapl2ctf7* triple homozygous (right) plants. (B) Alexander staining of anthers showing pollen viability in *AtCtf7^+/−^*, *Atctf7*, *Atwapl1-1wapl2*, *Atwapl1-1wapl2Ctf7^+/−^* and *Atwapl1-1wapl2ctf7* plants. Viable pollen stain red, while nonviable pollen stain green. Size Bar = 10 µm (C) Seed set in *Atwapl1-1wapl2Ctf7^+/−^* and *Atwapl1-1wapl2ctf7* plants is lower than that observed in *Atwapl1-1wapl2* plants. Images shown represent the most common phenotypes observed.

Finally, we investigated the possible genetic interaction between *AtWAPL* and *AtCTF7* by crossing *Atwapl1-1wapl2* plants with plants heterozygous for a T-DNA insertion in *AtCTF7* [45]. We were particularly interested in determining if inactivation of WAPL can suppress the dramatic affect of *Atctf7* mutations. *AtCTF7* is an essential gene with *ctf7* mutations causing female gametophyte lethality [45]. Plants homozygous for *Atctf7* mutations can however be recovered at very low frequencies [46]; the plants are dwarf, completely sterile and display multiple developmental alterations (Figure 13A). PCR genotyping was used to first identify plants triple heterozygous for the three mutations and then *Atwapl1-1wapl2ctf7^+/−^* plants were identified in F2 populations of several different crosses. *Atwapl1-1wapl2ctf7^+/−^* plants resembled *Atwapl1-1wapl2* plants, displaying relatively normal vegetative growth and reduced fertility (Figure 13C). *Atwapl1-1wapl2ctf7^+/−^* anthers (n = 16) produce on average 234±18.2 pollen and 41% (n = 1642) of the pollen produced was not viable (Figure 13B). Likewise, 43% of the ovules in siliques (n = 21) of *Atwapl1-1wapl2ctf7^+/−^* plants abort prior to fertilization and 52% of the seed produced (n = 2036) is shrunken and shriveled. Ultimately *Atwapl1-1wapl2ctf7^+/−^* plants produce on average17.9±3.3 viable seeds per silique (n = 23).

Plants homozygous for mutations in all three genes (*Atwapl1-1wapl2ctf7*) were readily obtained from selfed *Atwapl1-1wapl2Ctf7^+/−^* plants. The vegetative growth of *Atwapl1-1wapl2ctf7* plants is relatively normal, with the growth rate and overall size of the plants resembling that of wild type (Figure 13A). Further, while *Atctf7* plants are completely sterile, *Atwapl1-1wapl2ctf7* plants produce some viable pollen and seed (Figure 13B, C). *Atwapl1-1wapl2ctf7* plants produce 47±15.5 viable pollen/anther (n = 16) and approximately 10.8±4.3 normal seeds/silique (n = 23). Fewer ovules appear to be fertilized in *Atwapl1-1wapl2ctf7* plants; however those that are fertilized develop into viable seed.

## Discussion

In this study we investigated the role(s) of WAPL in *Arabidopsis*. Unlike other organisms that have been studied to date, *Arabidopsis* contains two active copies of *WAPL*. The *Arabidopsis WAPL* genes are highly similar and display similar transcriptional patterns. Mutations in each individual gene have no apparent effect, suggesting that the genes share overlapping functions. Plants double homozygous for the *Atwapl1-2* and *Atwapl2* mutations display normal vegetative growth and development and a modest reduction in fertility that results primarily from early ovule abortion. The presence of transcripts 3′ to the *Atwapl1-2* T-DNA insert, which is located in exon one, combined with the weak phenotype suggests that the insert may not totally disrupt splicing and a partially functional version of the protein may be produced in *Atwapl1-2* plants.

Plants containing the *Atwapl1-1wapl2* double mutant combination grew slightly slower than wild type and exhibited a greater reduction in fertility, which results from defects in both male and female meiosis. Mitotic alterations were also observed in some *Atwapl1-1wapl2* root tip cells, but these alterations did not have a noticeable impact on root growth or patterning. *AtWAPL1* transcripts are not present in *Atwapl1-1wapl2* plants. Low levels of truncated *AtWAPL2* transcripts are produced in *Atwapl2* plants raising the possibility that a truncated, partially functional version of *AtWAPL2* may be produced. If a truncated protein is produced it would be missing a minimum of 136 amino acids from the N-terminus of the protein (Figure S1), including a stretch of 25/55 highly conserved amino acids.

WAPL was first identified in *Drosophila*, where mutations typically cause embryo lethality [37]. However, a few “escapers” are able to develop into adults with wings that are abnormally separated. Neuroblasts of *Drosophila wapl* mutants arrest at metaphase with most chromosomes displaying prolonged cohesion. *Wapl* is also an essential gene in mice [47]. *Wapl* ^−/−^ mice were not obtained in experiments where Cre recombinase and “floxed” *Wapl* were used to generate null alleles [47]. Mouse embryonic fibroblasts in which a floxed *Wapl* locus was deleted displayed altered transcriptional patterns and contained chromosomes with hyper-condensed heterchromatin that failed to segregate properly at anaphase, ultimately leading to cellular arrest [47]. Reduction of Wapl in HeLa cells using siRNA blocked the dissociation of cohesin from chromosomes during mitotic prophase and delayed the resolution of sister chromatids, resulting in the accumulation of prometaphase-like cells [16], [17]. While most *Wapl* depleted cells eventually entered anaphase and separated their chromosomes, the cells ultimately arrested. In contrast, *Wpl/Rad61* is a nonessential gene in yeast. The growth of *wpl/rad61* mutants is indistinguishable from wild type; however, the mutants are sensitive to DNA damaging agents and show alterations in cohesin dynamics [48].

Similar to the situation in yeast, mutations in Arabidopsis *WAPL* do not have a significant impact on growth. Approximately 20% of *Atwapl1-1wapl2* root tip cells display altered mitotic figures, including the presence of “sticky chromosomes” at metaphase and chromosome bridges, lagging chromosomes and possibly chromosome fragments at telophase (Figure 11 D–F). However, most cells undergo normal division and cohesin complexes appear to be removed normally, including in cells that displayed mitotic defects (Figure 12). We cannot, however rule out the possibility that low levels of cohesin remain on the “sticky” mitotic chromosomes. Given that WAPL seems to play similar roles in controlling the interaction of cohesin with the chromosomes in all organisms studied to date, it is not clear why WAPL is an essential protein in flies and vertebrates, but not yeast and possibly plants. Further studies are required to address this question.

### AtWAPL is required for the prophase release of cohesin from meiotic chromosomes

Our results show that while AtWAPL is not critical for nuclear division in somatic cells, it is required for the proper release of cohesin from meiotic chromosomes during prophase. Most *Atwapl1-1wapl2* male meiocytes observed at metaphase I/early anaphase I contained “sticky chromosomes” that displayed strong SYN1 labeling. SYN1 is undetectable on the chromosomes of wild type meiocytes beginning at pro-metaphase I [44]. The formation of chromosome bridges at anaphase I and ultimately mis-segregated chromosomes at telophase I is likely due to the prolonged presence of chromosome arm cohesin in *Atwapl1-1wapl2* meiocytes. While some *Atwapl1-1wapl2* metaphase II chromosomes showed faint cohesin signals, the majority did not. This suggests that arm-associated cohesin complexes normally removed by WAPL during prophase are instead removed during telophase I/interphase II in the mutant, potentially through the action of separase. Although we did not specifically analyze meiosis in megasporocytes, the fact that a relatively large number of female gametophytes arrest at FG1 or FG2 suggests that inactivation of *AtWAPL* affects both male and female meiosis. Little is known about the role of WAPL in meiosis. *Drosophilia wapl* mutants exhibit meiotic alterations, specifically in the segregation of nonexchange X chromosomes and the loosening of adhesion between sister chromatids in heterochromatic regions [37]. In budding yeast inactivation of *Wpl* does not appear to affect spore formation and viability [49].

The chromosomal alterations we observe during meiosis in *Atwapl1-1wapl2* plants resemble those caused by depletion of WAPL during mitosis in human cell cultures and flies. Depletion of Wapl in human cell lines blocks the removal of cohesin during prophase resulting in poorly resolved sister chromatids [16]. Likewise, mitotic chromosomes in *wapl* flies also show prolonged arm cohesion that delay/block the resolution of sister chromatids at anaphase [37]. While yeast *wpl/rad61* cells display increased steady-state levels of cohesin, Wpl/Rad61 does not play a critical role in the removal of cohesin complexes during mitotic prophase [11], [13]. Rather, most mitotic cohesin complexes are removed from yeast chromosomes at anaphase by separase.

Interestingly, 65% of *Atwapl1-1wapl2* meiocytes contained more than the expected ten centromere signals at metaphase I/anaphase I. This suggests that while the removal of arm cohesin is delayed, centromere cohesion either is not established properly or is prematurely released. The aggregation of centromere sequences we observe during prophase indicate that there are alterations in heterchromatin structure, suggesting that meiotic chromosome centromere cohesion may in fact not form properly in the mutant. This is similar to the situation in Drosophila *wapl* neuroblasts in which the largely heterochromatic chromosomes 4 and Y display a precocious loss of cohesion, while the other chromosomes maintain arm cohesion and arrest at prometaphase [37]. Finally, Wpl appears to be important for controlling chromosome condensation in budding yeast where inactivation of *Wpl* results in increased compaction of chromosome arms in S/G2 [49]. Our results show that inactivation of *AtWAPL* results in the aggregation of heterochromatin regions in particular centromeres. Therefore, WAPL plays a common role in controlling chromosome structure.

### *Atwapl* mutations suppress lethality and restore partial fertility to *Atctf7* plants

We show here that *AtWAPL* mutations suppress the lethality associated with *ctf7* mutations in *Arabidopsis*. This is similar to similar to the situation in yeast [13], [20], [21], [23], [24]. Inactivation of *AtCTF7* results in embryo lethality [45]; however for reasons that are not understood, homozygous *Atctf7* mutant plants can be obtained at very low frequencies [46]. *Atctf7* plants are dwarf, exhibit severe developmental abnormalities and are completely sterile. They also display mitotic defects, alterations in double strand break repair and the premature dissociation of cohesin from meiotic chromosomes, which leads to the early separation of sister chromatids [46]. Plants triple homozygous for the *Atwapl1-1wapl2ctf7-1* mutations display normal vegetative growth and produce small numbers of viable seed. The growth rate and overall size of *Atwapl1-1wapl2ctf7-1* plants is similar to that of wild type, indicating that inactivation of *AtWAPL* suppresses most, if not all of the effects associated with CTF7 inactivation in somatic cells. Furthermore, inactivation of WAPL restores some fertility to *Atctf7-1* plants. The overall fertility of *Atwapl1-1wapl2ctf7-1* plants is significantly lower than that of *Atwapl1-1wapl2* plants but similar to that of *Atwapl1-1waplCtf7^+/−^* plants. Therefore, meiotic chromosomes are much more sensitive to the level and distribution of cohesin than somatic cells in plants.

Our results indicate that AtWAPL most likely functions during meiosis in a manner similar to that proposed for Wapl in mitotic cells in vertebrates. Prior to DNA replication cohesin has been shown to bind the chromatin in a reversible manner that is normally not able to establish sister chromatid cohesion [13], [16], [17], [23], [24], [50]. This reversible binding is controlled, in part, through interactions between Wapl, Pds5 and the cohesin complex. Stable cohesin binding to the chromosomes and the establishment of cohesion, which occurs during DNA replication, involves the inactivation of this Wapl-dependent anti-establishment activity through the Eco1/Ctf7-dependent acetylation of critical lysine residues in SMC3 [20]–[23], [51]. In animal cells, acetylation of SMC3 facilitates the recruitment of sororin and displacement of Wapl to help create a stable cohesin complex [15], [18]. A sororin ortholog has not been detected in yeast where SMC3 acetylation appears to directly inactivate the Wpl releasing activity and result in tight binding of cohesin to the chromosomes [22], [24], [52].

Most closely related to our work are studies in vertebrate cells that have shown that Wapl is involved in the non-proteolytic removal of cohesin from the arms of mitotic chromosomes as part of the prophase pathway [25]. This process, which involves the mitotic kinases Polo-like kinase (Plk1) and Auora B [17], [28], [41], [53], [54], involves opening of the cohesin ring at the junction between SMC3 and the SCC1 WHD [26], [27]. Plk1 and Auora B have been shown to phosphorylate multiple sites on Sororin, which leads to the disassociation of Sororin from acetylated cohesin complexes [55]. SA2/SCC3 is also phosphorylated by Plk1 [28], which likely also alters the interaction of Wapl with cohesin.

Finally, structural studies on Wapl from fungi and human have generated partial structures of Wapl, which have provided further insights into how Wapl exerts its' anti-maintenance activity and the residues important for interactions between Wapl, Pds5 and cohesin [38], [39], [56]. A number of features are shared between the fungal and human Wapl proteins; however, several structural and mechanistic differences were also identified. These structural differences are likely related to the fact that Sororin plays an important role in the Wapl-dependent opening of the cohesin ring in vertebrates but not in yeast.

The removal of cohesin from meiotic chromosomes in *Arabidopsis* involves a prophase step [57], which we show here is dependent on WAPL. This suggests that the process may also involve the phosphorylation of SCC3. Further studies are required to test this hypothesis and determine if an Aurora or Polo-like kinase is involved in this process. Likewise, a sororin ortholog does not appear to be present in the *Arabidopsis* genome, suggesting that acetylation of SMC3 may directly interfere with WAPL binding in plants. However, further experiments are necessary to determine if *Arabidopsis* SMC3 is actually acetylated by CTF7 and if this affects WAPL binding. Furthermore, while five potential PDS5 orthologs are present in the *Arabidopsis* genome, a role for the proteins in controlling sister chromatid cohesion has not yet been established. Therefore, additional studies are needed to further characterize the roles of WAPL, PDS5 and CTF7 in plants and further define the specifics of how they control the association of cohesin with chromatin. These studies will help us to better understand the apparent differences in how cohesin interacts with chromosomes in meiotic and somatic cells and determine the specific reason(s) meiotic and mitotic plant cells respond so differently to *Atwapl* mutations.

## Materials and Methods

### Plant material and growth conditions

*Arabidopsis thaliana*, Columbia ecotype, was used for crossing, transcript analysis and microscopic studies. Plants were grown in Metro-Mix 200 soil (Scotts-Sierra Horticulture Products; http://www.scotts.com) or on germination plates (Murashige and Skoog; Caisson Laboratories; www.caissonlabs.peachhost.com) in a growth chamber at 22°C with a 16-h-light/8-h-dark cycle. *Arabidopsis* T-DNA lines were obtained from *Arabidopsis* Biological Resource Center. Leaves were collected from rosette-stage plants grown on soil and used for DNA isolation and genotyping. Approximately 24 d after germination, buds were collected and staged for microscopy studies. For transcript analysis all samples were harvested, frozen in liquid N_2_, and stored at −80°C until needed. A description of the molecular characterization of *AtWAPL1* and *AtWAPL2* is provided in Text S1. Sequences of primers used in this study are given in Table S1.

### Chromosome analysis and immunolocalization

Male meiotic chromosome spreads were performed on floral buds fixed in Carnoy's fixative (ethanol∶chloroform∶acetic acid: 6∶3∶1) and prepared as described previously [58]. Chromosomes were stained with using DAPI and observed under Olympus BX51 epifluorescence microscope system. Images captured using a Spot camera system and processed using Adobe Photoshop.

In order to study mitosis *Arabidopsis* seeds were sterilized and plated on MS agar plates. Seven days after plating the root tips from the seedlings were excised, fixed and prepared as described previously [58], with the exception that digestion was conducted for 15 min.

Immunolocalization studies were performed on 4% paraformaldehyde fixed cells as previously described [59]. Meiotic stages were assigned based on the chromosome structure and morphology as well as the developmental stages of the surrounding anther cells. Primary antibodies (1∶500 dilutions) used in this study (SYN1, SMC3, ASY1, ZYP1, β-tubulin) have been described [43], [44], [60], [61]. The slides were incubated overnight at 4°C, and then washed for 2 h with eight changes of wash buffer. The slides were then incubated overnight with Alexa 488 labeled goat anti-rabbit secondary antibody (1∶500) or Alexa Fluor 594 labeled goat anti-mouse secondary antibody (1∶500) overnight at 4°C and again washed and stained with DAPI.

FISH was conducted on inflorescences fixed in Carnoy's solution for 1 h at room temperature after replenishing the fixative. FISH was performed on meiotic spreads as previously described [62], [63] with the following change: samples were treated with a solution of freshly prepared 70% formamide in 2× SSC for 2 min at 80°C and dehydrated through a graded ethanol series (70%, 90%, 100%) of 5 min for each incubation at −20°C. The slides were then dried at room temperature before adding the probe. The 180-bp pericentromeric repeat [64] was amplified, purified, labeled with Roche High Prime fluorescein and was used at a concentration of 5 ugml^−1^. Telomere-repeat sequences were detected by hybridization with the 5′-end fluorescein isothiocyanate-labeled oligonucleotide probe, (CCCTAAA)_6_ at 5 ugml^−1^. Slides were counterstained with DAPI and observed under epifluorescence microscope as described above.

### Expression analyses

Total RNA was extracted from stems, buds, roots, leaves and siliques of wild-type plants to examine *WAPL* expression patterns, and from inflorescences of wild-type, *Atwapl1-1wapl2* and *Atwapl1-2wapl2* plants to measure *WAPL* transcript levels in mutant plants. Total RNA was extracted from with the Plant RNeasy Mini kit (Qiagen, Hilden, Germany), treated with Turbo DNase I (Ambion) and used for cDNA synthesis with an oligo (dt) primer and a First Strand cDNA Synthesis Kit (Roche). Real time PCR was performed with SYBR-Green PCR Mastermix (Clontech) and the amplification was monitored on a CFXsystems (Biorad). Expression was normalized against *β-Tubulin-2*. At least three biological replicates were performed, with two technical replicates for each sample. Primers used in this study are presented in (Table S1).

### Analysis of male and female gametophyte development and embryo development

Whole-mount clearing was used to determine the embryo phenotypes [65], [66]. Sliques from wild-type and mutant plants were dissected and cleared in Hoyer's solution containing lactic acid∶chloral hydrate∶phenol∶clove oil∶xylene (2∶2∶2∶2∶1, w/w). Embryo development was studied microscopically with a Olympus BX51 microscope equipped with differential interference contrast optics. Female gametophyte analysis was performed as described in [67]. Whole anther morphology was analyzed by staining with Alexander staining [68].

## Supporting Information

Figure S1 Clustal W multiple sequence alignment of WAPL protein family representatives. Black and gray shades indicate identical and similar amino acids, respectively. FGF motifs are highlighted in yellow for *Homo sapien Wapl*. The position of T-DNA insertion site in *AtWAPL2* is shown with an “*”.
(DOC)

Figure S2 Localization of ASY1 in wild type and *Atwapl1-1wapl2* mutant meiocytes. The distribution of ASY1 was similar between wild type and *Atwapl1-1wapl2* plants at zygotene (A, B) and pachytene (C, D). Size Bar = 10 um.
(PDF)

Figure S3 Localization of ZYP1 in wild type and *Atwapl1-1wapl2* mutant meiocytes. ZYP1 immunolocalization on pachytene stage meiocytes from wild type (A–C) and *Atwapl1-1wapl2* (D–L). Left panel indicates the DAPI stained chromosome. Middle panel shows green signal for ZYP1 and the right panel shows the merged DAPI and ZYP1 signals. Cells with discontinius labeling are shown in D–I and a cell with unsynapsed regions is shown in J–L. Size Bar = 10 um.
(PDF)

Figure S4 Spindle abnormalities observed in *Atwapl1-1wapl2* male meiocytes at metaphase I (A–D), anaphase I (E–H), and meiosis II (I–L). Size Bar = 5 um.
(PDF)

Figure S5 Embryo alterations observed in *Atwapl1-1wapl2* siliques. Embryo arrested at 1-cell stage with an abnormal suspensor (A). Abnormal four cell embryo (B). Normal appearing two cell embryo that is arrested/delayed (C). Two cell embryo with the abnormal divisional planes and suspensor (D). Normal appearing eight cell embryo that is arrested/delayed (E). Normal appearing dermatogen that is arrested/delayed (F). Normal appearing globular stage that is arrested/delayed (G). Normal appearing early heart stage embryo is arrested/delayed (H). Embryos shown in B, E, F, G and H were all observed in sliques with cotyledon staged embryos. Size bar = 10 µm.
(PDF)

Table S1 Primers used in this study. Sequences of primers used in this study are shown.
(PDF)

Text S1 Molecular characterization of *Atwapl* mutants. A description of the molecular analysis of the T-DNA insertion sites associated with *AtWAPL1* and *AtWAPL2* along with the corresponding genes is provided.
(DOCX)
