# Supplementary material for: Arabidopsis thaliana WAPL Is Essential for the Prophase Removal of Cohesin during Meiosis
Source: PLoS Genet. 2014 Jul 17;10(7):e1004497. doi: 10.1371/journal.pgen.1004497 (PMC4102442; doi:10.1371/journal.pgen.1004497)
Supplement: Figure S1 — Clustal W multiple sequence alignment of WAPL protein family representatives. Black and gray shades indicate identical and similar amino acids, respectively. FGF motifs are highlighted in yellow for Homo sapien Wapl. The position of T-DNA insertion site in AtWAPL2 is shown with an “*”. (DOC) [file pgen.1004497.s001.doc]

Drosophila 1 KEEKKLKPEAPPSRVLGRARKAVNYREVDEDERYPTPTKDLIIPKAGRQPGEVAATATLA

Drosophila 61 AASSEAFISSTFGSPGSEPSLPPPTSAPSATASTASQLPSASG--------GASN---PP
Human 1 -------MTSRFGKTYSRKGGNGSSKFDEVFSNKRTTLSTKWGETTFMAKLGQKRPNFKP

Drosophila 110 SASRTPEHPPIVLRISKGTSRLVSTDSEEPPSSSPAHHNQLNQLSVTEEEPPEREATEAA
Human 54 DIQEIPKKPKVE-EESTGDPFGFDSDDESLPV----------------------------

Drosophila 170 PSSRFRRWIICGSWRSISRRFF------------------------RGTQVPVTRTNEDE
Human 85 -SSKNLAQVKCSSYSESSEAAQLEEVTSVLEANSKISHVVVEDTVVSDKCFPLEDTLLGK

Drosophila 206 EEEEEEEDEEEEPPEINYCTVKISPDKPPKERLKLIIKTDVIRNAIAKAAAAAESRSEKK
Human 144 E--KSTNRIVEDDASISSCNKLITSDKV--------------ENF-----------HEEH

Drosophila 266 SRSKKHKHKQLLAAGSGAAPASGATPAEINSEFKTPSPHLALSEANSQQAQHTPSQLHQL
Human 177 EKNSHHIHKN--ADDSTKKPNAET---TVASEIKETNDTW-----NSQ-FGKRPE---SP

Drosophila 326 HQLHPQRGSAVISPTTRSDHDFDSQSSVLGSISSKGNSTPQLLAQAVQEDSCVIRSRGSS
Human 223 SEISPIKGSVRTG-LFEWDNDFE----------------------DIRSEDCILSLDSDP

Drosophila 386 VITSDLETSQHSSLVAPPSDIESRLESMMMTIDGAGTGAASAVPETPLQEDILA-VLRGE
Human 260 LLE------------MKDDDFKNRLENLNEAI----------------EEDIVQSVLRPT

Drosophila 445 VPRLNGNTDPEPTEEEDQQQQPKRATRGRGRKANNNVDVT----P-----PATETRTRGR
Human 292 NCRTYC-----------------RANKTKSSQGASNFDKLMDGTSQALAKANSESSKDGL

Drosophila 496 AKGTDATTAAISPPAGKRTTRGTRGSRKAEQEVDMEVDETAVATVP---ANEEQLEQ--A
Human 335 NQA---KKGGVS---CGTSFRGTVGRTRDYTVLHPS--CLSVCNVTIQDTMERSMDEFTA

*S.cerevisiae* 1 MRAY---------GKRGPVLR
Drosophila 551 TLPPRRGRNAAARAN-------NNNLASVNNNINKIAANLSAKAEASRLAEGGVAGGAAR
Human 387 STPADLGEAGRLRKKADIATSKTTTRFRPSNTKSKKDVKLEFFG-FEDHETGGDEGGSGS
*C.elegans* 1 MSSDA-----NSDDPFSKPIVRKRFQATLAQQG-IEDDQLPSV------

*S.cerevisiae* 13 TPFRS---NKGLPSSSDVEFSDDD-V-NSVIPDVSSTISSSIADHPIEGLLDEPRKAQDS
Drosophila 604 S-YGRKRKNQQVTQV----------LQQEPAPEEQE-----TPDAEE--EQPTPAKIPHT
Human 446 SNYKIKY--FGFDDLSESEDDEDDDCQVERKTSKKR-----TKTAPSPSLQPPPESND--
*C.elegans* 38 --------------------------RSSDSPDVPD-----TPDVPVNQLSSPPLSLPET

*S.cerevisiae* 68 SSSFDGANEKPSSQLDSKRNDQNVKIITSSDTSMAFMKD--------------EKLSAFN
*S.pombe* 1 MKRGKCKEKDNGLKRISSESEVWN
*AtWAPL1* 1 MIIVKLTAN----RICCSLLQLRRSYE--HFYIFV
Drosophila 646 DH---REH-SPDHDPD-----PDPDELSNNSNNSSLQHDGS-SSSPPPRDYKFKDKFKRT
Human 497 ------------------------------------------NSQDSQSGTN--NAENLD
*C.elegans* 67 LS---EGNAETNLSDD-----SEPEMLSQSST-SSLNRRMEDSAIDPSRGTR--KSQSRG

*S.cerevisiae* 114 FLDGSKAS---------------------------------------------KRKRRRT
*S.pombe* 25 FLDVTVSELNKQKRSPGQTVSKRLHKKQ-RVVSNPDLSLP----SSPVKQILRNGLQNSK
*AtWAPL1* 30 FLPEI-----------------P-------LFRFSHLKLFPKNLQIQR--LV-SAMMERT
*AtWAPL2* 1 MMERT
Drosophila 696 LTLDSQGAANAGAGGAAA-AT-PPESSGE---QRGAVKLA----TVAKRHLY-KHSWDAA
Human 513 FTEDLPGV-------PES-VKKPINKQG-DKSKENTRKIFSGPKRSPTKAVYNARHWNHP
*C.elegans* 116 FDYDPAGE---------R-TTAPVQKKKKDEIDMGGAKFFPKQ---EKKHVY-THKWTTE

*S.cerevisiae* 129 YQKHDANITSSIEPDVQDEDSITMHNEFESIRKIYNDINEFILKLPRADDDILN-KMLEN
*S.pombe* 80 YGSHKTGLERSASCSSIDAS----------------------------------------
*AtWAPL1* 63 YGRRKPGIPRTLSDSLNDSV----SQ------------TEYLSSSSSPDIEPIDYSLLPF
*AtWAPL2* 6 YGRRKPGMLND-------DV----SRA-----------EHIFPSSSSPELEPVDF-----
Drosophila 746 LEANGGGTNSDASNASASGV--GVAGA-------KDHLHHLAAG-----KSDGDFGDSPS
Human 564 DSEELPGPPVVKPQSV--TV-------------------RLS-------------SKEPN
*C.elegans* 162 EDDEDEKTI--S--SS--SN-------------------RY--------------SSRPN

*S.cerevisiae* 188 EMKMDDSIENNSIRTSKDKKYGKFRTILI-NKNKENEIMGEEVDQKANTLSLNN------
*S.pombe* 100 -ANHSSTTYR-EQRSYLMEEGLDTQPIVPREVSSG-----RELDSTNHTIG------TER
*AtWAPL1* 107 SSQESSSLWHSSSRSNFREDYPQNGGVVRRA-----------------KRVRNG------
*AtWAPL2* 39 STQESSCVWNYSSRSTFSDN----DFSEKRN-----------------KRPRNG------
Drosophila 792 SNNN-GSSSACSSASTLRGDSPALGKISRLAGKQGRSAAGASVGGTGATTGGGGATGGGG
Human 590 QKDD-GV---------FKAPAPPSKVIKTV------------------TIPTQPYQDIVT
*C.elegans* 183 Q---------------------PAV----S------------------ARPRQPVYATTS

*S.cerevisiae* 241 ----ADNSNAEKEGLTSTNHYNELKNMGDTIKYQDDIEFLLSNSKSNDNTTVPINEYFKK
*S.pombe* 147 AFLIEEDV--SEDDEIQMKSIHELRFAGEQQRIVDEIEYLVDGVTFSGNSSASRYLSL--
*AtWAPL1* 144 -----------AEAAAFTSTLLEAQEFGELMEHEDEVNFALDGLRKG-HQLRIRRASL--
*AtWAPL2* 72 -----------GGGFGSNSTLMEAQEFGELIENEDEVNFALDGLKKG-HKVRIRRAAL--
Drosophila 851 PIRVDRKTKDYYPVVRNVKTAHQIQEIGEYQEMDDDVEYILDALQPH-NPPATRCLSA--
Human 622 ALKCRREDKELYTVVQHVKHFNDVVEFGENQEFTDDIEYLLSGLKST-QPLNTRCLSV--
*C.elegans* 200 -TYSKPLASGYGSRVRHIKEANELRESGEYDDFKQDLVYILSSLQSSDASMKVKCLSA--

*
*S.cerevisiae* 297 LLNLSLMIINDEEFFQYAKRYFKKEI----------IKLSFAQFRSDFPELILLQGYLLH
*S.pombe* 203 ---IGIAEK---MFDNSFRLCLKSIRDVFLRIFEEID---PK---DTLHTFL--QIYIFA
*AtWAPL1* 190 ---SSLLSI---CASQHQRRSLRAQGIS-QSIIDAILVLSLDDIPSNLAAAT--LFFALT
*AtWAPL2* 118 ---SSLLSI---CESQYQRRSLRALGIS-QSIIDAILGLCLDDIPSNLAAAT--LFFVLT
Drosophila 908 ---LQLAAK---CMMPAFRMHVRAHGVV-TKFFGALSDAN-KDLSLGLCTSA--IMYILS
Human 679 ---ISLATK---CAMPSFRMHLRAHGMV-AMVFKTLDDSQ-HHQNLSLCTAA--LMYILS
*C.elegans* 257 ---ISLAKK---CVSPDFRQFIKSENMT-KSIVKALMDSP-EDDLFALAAST--VLYLLT

*S.cerevisiae* 347 K-------VSESQSDFPPSFDNFSIELSKDDGKIRTK---KNKHI---------------
*S.pombe* 249 TMANEMDCMSSLLDAYSNNV-KLLLQT----AITLEPQVPVSILAK--SLPK--------
*AtWAPL1* 241 ADGQDEHFMES-----PKCI-KFLIKLLKPVIVTSTEGKPRNIGFKLLSLLKDVDAARDP
*AtWAPL2* 169 TDGQDDHFMES-----PNSI-KFLVKLLRPVVSASTKVKPRNIGSRLLSIIKDVDAARDA
Drosophila 958 QEGLNMDLD-------RDSL-ELMINLLEADGVGGS------------------------
Human 729 RDRLNMDLD-------RASL-DLMIRLLELEQDASS------------------------
*C.elegans* 307 RDFNSIKID-------FPSL-RLVSQLLRIEKFEQR------------------------

*S.cerevisiae* 382 KKLSHLNFEDFLRKTQF--------KTGLYYSLSLWEMHGNLSLD---IIKRISILASNK
*S.pombe* 294 ---------SVKGAVQEFVIKA---ELTFSFSNESLASSDSISLAAIALMKTSSGVFAES
*AtWAPL1* 295 VKMDDPSSSDILSRVQELLVNCKEMRLNDSYITETTRP----------------------
*AtWAPL2* 223 ASMHDLSSCDIIDRAQEILVNCKELRLIDSYKIERMRP----------------------
Drosophila 986 TETGHPDRAGYDRNKQKVRELCEEIKA--QGKGTHLNV----------------------
Human 757 AKLLN--EKDMNKIKEKIRRLCE------TVHNKHLDL----------------------
*C.elegans* 335 PEDK---DKVVNMVWEVFNSYIEKQEVGGQKVSFDMRK----------------------

*S.cerevisiae* 431 DLFSRHVKTFI----PLLEK-----LITASEFCHMYIEQPEMFDSL-ISNLNNQFKDMLD
*S.pombe* 342 ELFTELINLLIEKSYPILKENDGSNNFLLHALCSSLEKFTDFQGSEKIQKVSQILSSKL-
*AtWAPL1* 333 EL-----------------STKWVALLAMERACVSKISFDDTSGSVKKT--GGNFKEKLR
*AtWAPL2* 261 EL-----------------STKWVALLVMEKACLSKISFDDTSGTVKKS--GGMFKEKLR
Drosophila 1022 -------------------DSLTVGTLAMETLLS--------LTSK-RA--GEWFKEDLR
Human 787 -------------------ENITTGHLAMETLLS--------LTSK-RA--GDWFKEELR
*C.elegans* 370 -------------------ESLTPSSLIIEALVF--------ICSRSVN--DDNLKSELL

*S.cerevisiae* 481 DDSLIKILILLTNMEVHNYTLWKEADMIFQSSMNTILESIHPLTDAKVDNILLHLGLCLN
*S.pombe* 401 ---------QILIDEHNETNSPK-------------------IDD---------------
*AtWAPL1* 374 ELGGLDAVLEVVMDCHAVMERWVEYDALSV----------QEKKDNLHKQSLMLLLKCLK
*AtWAPL2* 302 ELGGLDAVFDVVMDCHTVMESWVTHDTLSV----------EDIKDDLNKQSLMLLLKCLK
Drosophila 1052 KLGGLEHIIKTISDFCSPVIACDTE--ID--------------WQPTLLDNMQTVARCLR
Human 817 LLGGLDHIVDKVKECVDHLSRDED--------------------EEKLVASLWGAERCLR
*C.elegans* 401 NLGILQFVVAKIETNVNLIADNAD--------------------D---TYSILILNRCFR

*S.cerevisiae* 541 ICSREN------SRLK---------LDGKLWYDM-KTIFVKMIR-----DGSDTENR---
*S.pombe* 418 ------------------------------------------------------------
*AtWAPL1* 424 IMENATFLSTDNQNHLLGFKKCLGSHDSRMSFTELTISVIKMLSGLHLRGGFSSPNTNNV
*AtWAPL2*  352 IMENATFLSTENQIHLLRLNKSMGSHESRLSFTELMISVIKILSGLQLRAHRNEKHPHPQ
Drosophila 1096 VLENVTQHNEANQRYMLTSGQGKAVE-----------TLCQLYR----------------
Human 857 VLESVTVHNPENQSYLIAYKDSQLIV-----------SSAKALQ----------------
*C.elegans* 438 ILESSSVFHKKNQAFLISHRSNILIS-----------SLAKFLQ----------------

*S.cerevisiae* 577 -----------------------------------------------LVQGLFYLNFSFL
*S.pombe* 418 ------------------------------------------------------------
*AtWAPL1* 484 NSHYSNGGNHDSVLEANRKVTNEVVTISSDTYSTVGSISTRNGSVSQRSQSIIHLDFSPT
*AtWAPL2* 412 -PHL-----------ASAVKKGFVTIISSDTCSTTGFSSIKSLSVSKRNQSAFLVGCSTT
Drosophila 1129 ------------------------------------------------------------
Human 890 ------------------------------------------------------------
*C.elegans* 471 ------------------------------------------------------------

*S.cerevisiae* 590 IKQRKENS------------------NLDPGELNLLLVELEAFKSETSQFNEGISNKIEI
*S.pombe* 418 ------------------------------------------------------------
*AtWAPL1* 544 SMSGSQSSVSGNEPTTSKTRVGSTISGSFAGRLASLGSDIARTTLRTTQAGEPICKKFGE
*AtWAPL2* 460 PKPGSQSSVMSTIDHCTLTTTAGSNTGSFAGRLASLGSGISRSKTRTSQTRESSCKKVEN
Drosophila 1129 --------------LC------------------------SRQIMLHPSDG---------
Human 890 --------------HC------------------------EELIQQYNRAEDSICL----
*C.elegans* 471 --------------VI------------------------LDRVHQLA------------

*S.cerevisiae* 632 ALN--------------Y-------------------LKSIY------------------
*S.pombe* 418 ------------------------------------------------------------
*AtWAPL1* 604 FAPPEESEDPFAFDLEDYKPSKWAVVSVNQKKSRAQKKKGCYKQSKDESLYQLFSSQEES
*AtWAPL2* 520 FASFEDSQDPFSFDLEDSGPSRWAVGK--QKKSKGQKRKGSYRDKKDERSLQLFSSQEE-
Drosophila 1142 ------------------------------------------------------------
Human 908 ---------------ADSKPL--------------------------------------- *C.elegans* 481 ------------------------------------------------------------

*S.cerevisiae* 641 ------------------------------------------TSERITI-----------
*S.pombe* 418 ----------------------------------TVVHACSEKLLRTLIQTVNSNSDHAL
*AtWAPL1* 664 SNHRLNSQEESSNRDCSTSLQPSHCTNDIDEECLCLLFDCLLTAVKVLMNLTNDN-----
*AtWAPL2* 577 SNHGLNSQEESSDRDHHVTEQPS-LTYDIDKGCLCLLSDCLLTAVKVLMNLTNGN-----
Drosophila 1142 --G-----------------------GSNKEHPGVAMRELLVPVLKVLINLTHTFNEAQP
Human 914 -PH-----------------------QNVTNHVGKAVEDCMRAIIGVLLNLTNDN-----
*C.elegans* 481 ------------------------------EEEVKKYISCLALMCRLLINISHDN-----

*S.pombe* 444 AVSK-----------------SEVPLFAYKILQKFSNF-----------SSDDETTRELI
*AtWAPL1* 719 VVGCRQVGGCRGLESMAELIARHFPSFTRS--QLFSEMEKTGSSHQKKDKYLTDQELDFL
*AtWAPL2* 631 SVGCREVAACGGLESMAELVVGHFPSFTRS--PLYSQMESGT--CHQKDKHLTDQELDFL
Drosophila 1177 SLGAELLGQRGDVVETS------FRLLLLS--AN---------------YIPDQCVFELS
Human 945 EWGSTKTGEQDGLIGTA------LNCVLQV--PK---------------YLPQEQRFDIR
*C.elegans* 506 ELCCSKLGQIEGFLPNA------ITTFTYL--AP---------------KFGKENSYDIN

*S.pombe* 476 ILILGLLLGLVEESHEFIQTITHVEVSFGA-------------------SALDVLISFYQ
*AtWAPL1* 777 VAILGLLVNLVERDGVNRSRLASASVPITKPEELQESEQ----------EMIPLLCSIFL
*AtWAPL2* 687 VAILGLLVNLVEKNGINRSRLAAASVPITNPEGLQDSEQ----------DMIPLLCSIFL
Drosophila 1214 ILVLTLLINLCMHTVPNRAALMQAAAPAEYVAD--------NPPAQGSVSALQALLEYFY
Human 982 VLGLGLLINLVEYSARNRHCLVNMETSCSFDSSICSGEGDDSLRIGGQVHAVQALVQLFL
*C.elegans* 543 VMMTSLLTNLVERCNANRKVLIAQTVKMVI-----PGHD------VEEVPALEAITRLFV

*S.pombe* 517 KNESIVEISGYVV---MILSHCFLNDPKA-FAQLKPLISQF--------YESLHK--FKN
*AtWAPL1* 827 TNQGSAETKEE----------------------------------------------TTT
*AtWAPL2* 737 TNKGSADTKDE----------------------------------------------TST
Drosophila 1266 KCEELARLVEKNTDAFLESN-------------------------------EKGKKKQEE
Human 1042 ERERAAQLAESKTDELIKDAPTTQHDKSGEWQETSGEI-QWVSTEKTDGTEEKHKKEEED
*C.elegans* 592 YHESQAQIVDADLDRELAFDVC--------------------------------------

*S.pombe* 563 FHLKLKE---------ELMMMGSNGLAIVSIID----ELHKSLQDYLRSDLVK-------
*AtWAPL1* 841 FTLDDEEAVLEGEKEAEKMIVEAYSALLLAFLSTESRSIRNSIKDYLPKRNLAILVPVLE
*AtWAPL2* 751 FTLDDEEAVLESEKEAEKMIVEAYSALLLAFLSTESRSIRNAIRDYLPKRDMAILVPVLD
Drosophila 1295 VEETVNNLVQRAGHHMEHTLKGSYAAILVGNLIADNELYESVVRRQLRGNSFKEIIGVLE
Human 1101 EELDLNKALQHAGKHMEDCIVASYTALLLGCLCQESPINVTTVREYLPEGDFSIMTEMLK

*AtWAPL1* 901 RFVAFHMTLNMIPPE------THKAVMGVIESCKSP------------------------
*AtWAPL2* 811 RFVAFHTTLDMIPPE------THKVVMEVIESCKLP------------------------
Drosophila 1355 KYHTFMNLTSSLEAAFVAHMKSTKRIIDNFKKRDYIYEHSDEHDNPLPLNLETTAQVLAV
Human 1161 KFLSFMNLTCAVGTTGQK---SISRVIEYLEHC---------------------------

Drosophila 1415 GADASHAASSSTTVASASAPSSTSATGTTRAPRVYKTYSSHR
