# Supplementary figures and images for: Arabidopsis thaliana WAPL Is Essential for the Prophase Removal of Cohesin during Meiosis
Source: PLoS Genet. 2014 Jul 17;10(7):e1004497. doi: 10.1371/journal.pgen.1004497 (PMC4102442; doi:10.1371/journal.pgen.1004497)

WT

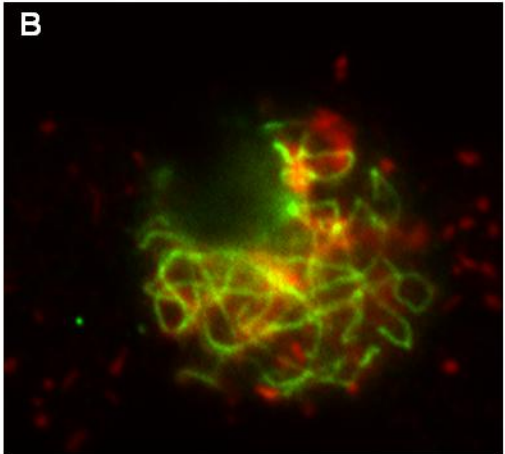

*Atwap11-1wap12*

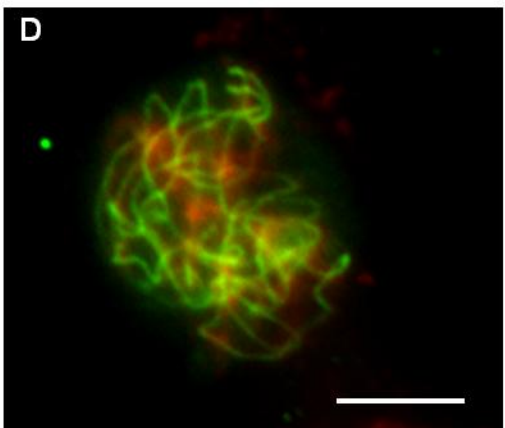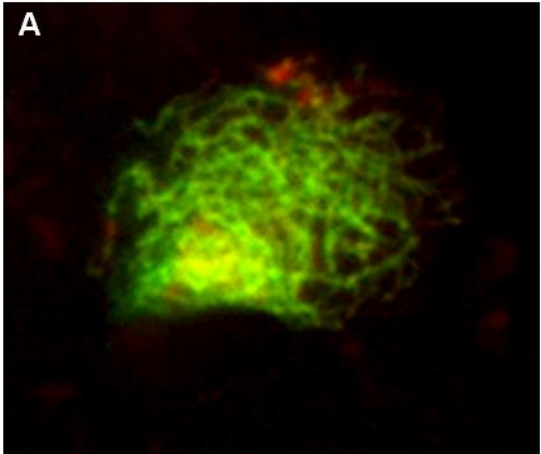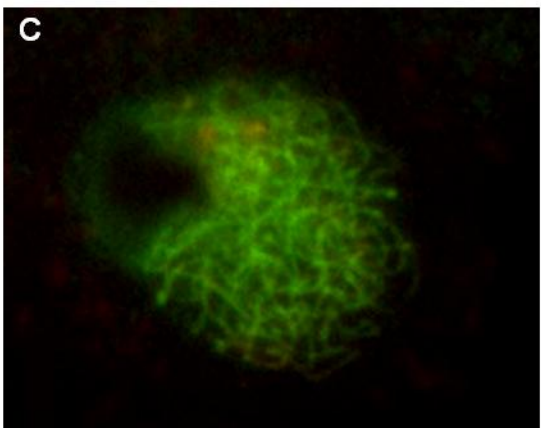

Supplement: Figure S2 — Localization of ASY1 in wild type and Atwapl1-1wapl2 mutant meiocytes. The distribution of ASY1 was similar between wild type and Atwapl1-1wapl2 plants at zygotene (A, B) and pachytene (C, D). Size Bar = 10 um. (PDF) [file pgen.1004497.s002.pdf]

DAPI

ZYP1

MERGED

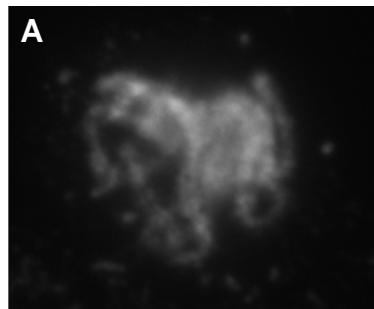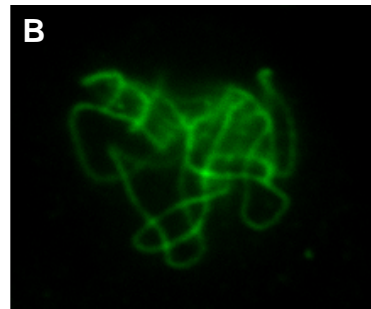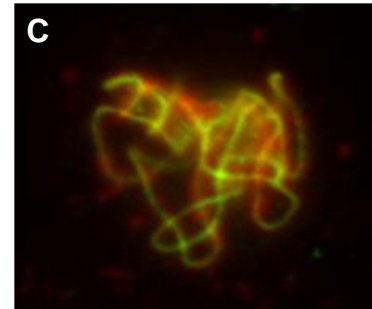

WT

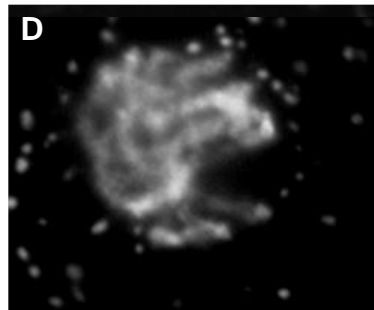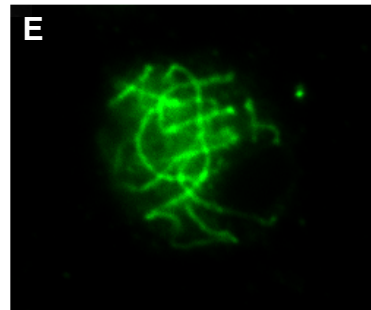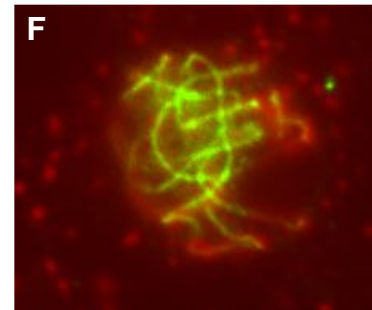*Atwap1.1wap12*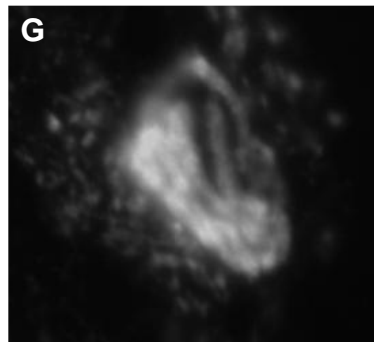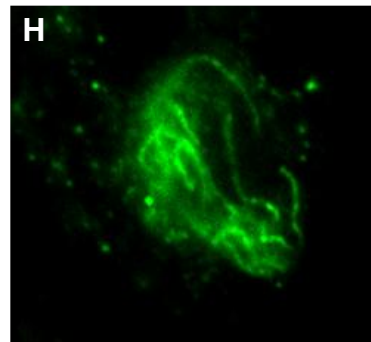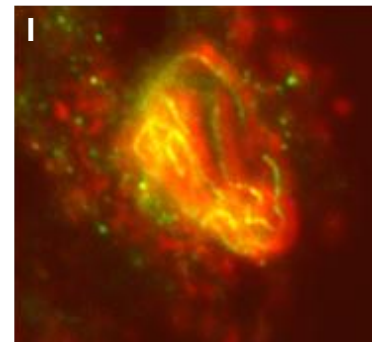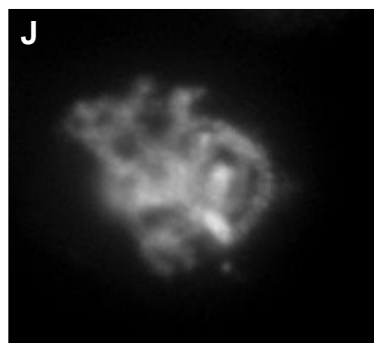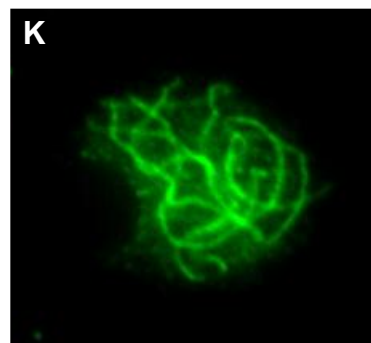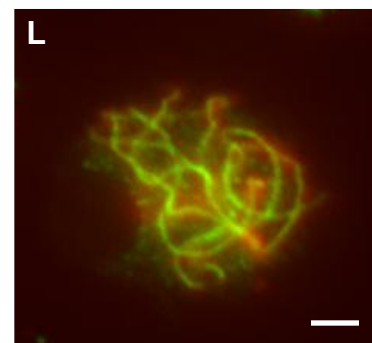

Supplement: Figure S3 — Localization of ZYP1 in wild type and Atwapl1-1wapl2 mutant meiocytes. ZYP1 immunolocalization on pachytene stage meiocytes from wild type (A–C) and Atwapl1-1wapl2 (D–L). Left panel indicates the DAPI stained chromosome. Middle panel shows green signal for ZYP1 and the right panel shows the merged DAPI and ZYP1 signals. Cells with discontinius labeling are shown in D–I and a cell with unsynapsed regions is shown in J–L. Size Bar = 10 um. (PDF) [file pgen.1004497.s003.pdf]

*Atwap1-1wap12*

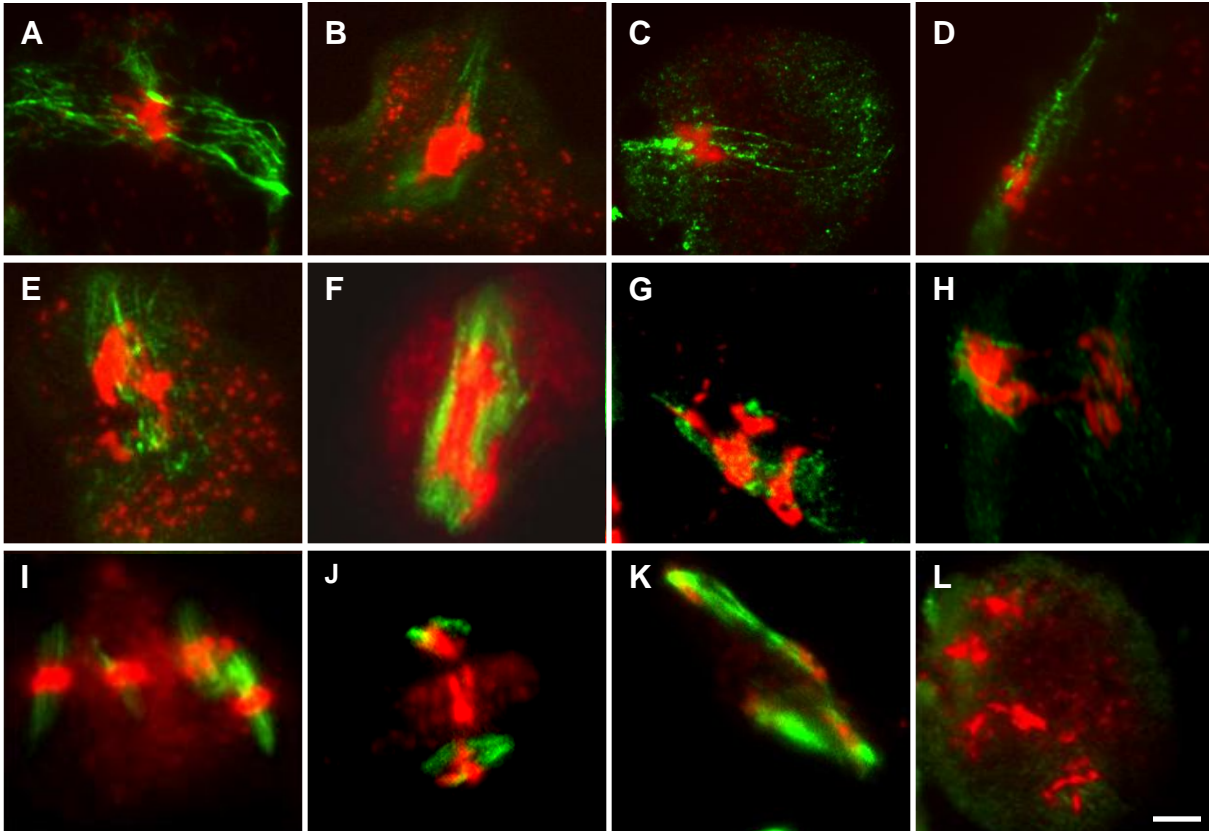

Supplement: Figure S4 — Spindle abnormalities observed in Atwapl1-1wapl2 male meiocytes at metaphase I (A–D), anaphase I (E–H), and meiosis II (I–L). Size Bar = 5 um. (PDF) [file pgen.1004497.s004.pdf]

***Atwap1.1wapl2* cleared silique**

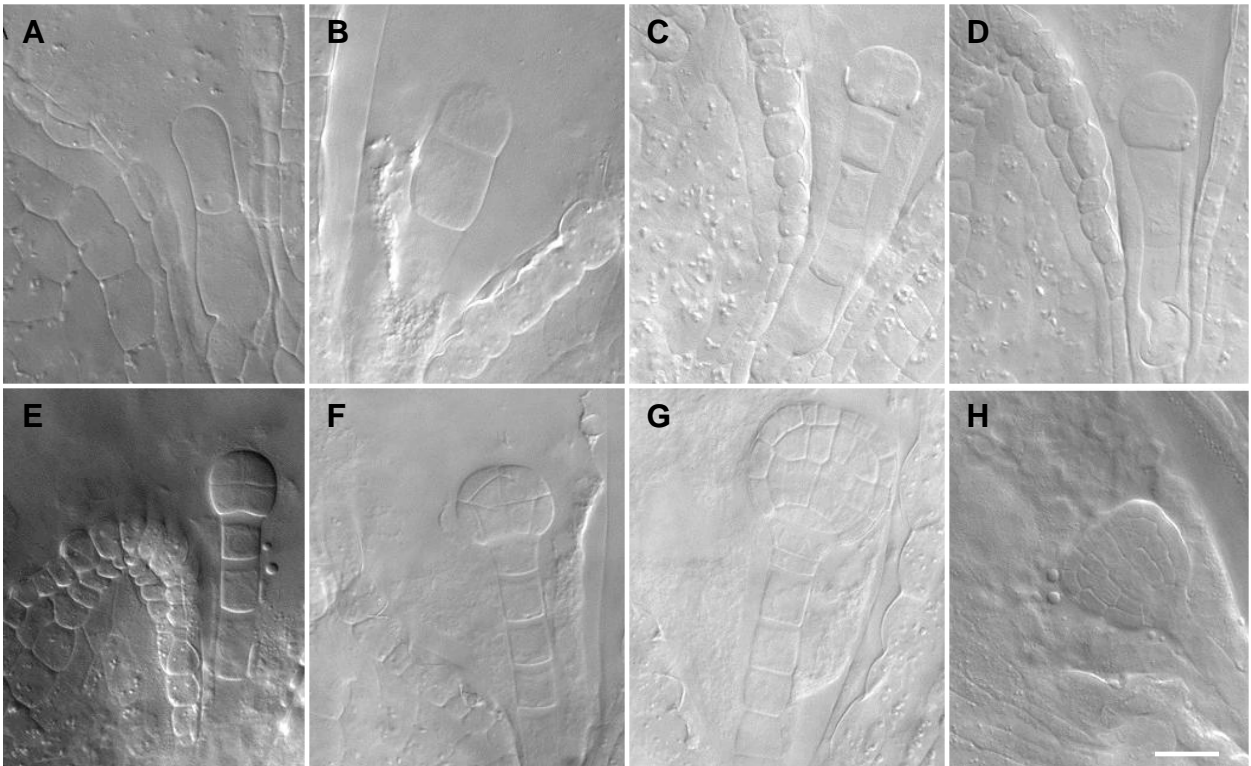

Supplement: Figure S5 — Embryo alterations observed in Atwapl1-1wapl2 siliques. Embryo arrested at 1-cell stage with an abnormal suspensor (A). Abnormal four cell embryo (B). Normal appearing two cell embryo that is arrested/delayed (C). Two cell embryo with the abnormal divisional planes and suspensor (D). Normal appearing eight cell embryo that is arrested/delayed (E). Normal appearing dermatogen that is arrested/delayed (F). Normal appearing globular stage that is arrested/delayed (G). Normal appearing early heart stage embryo is arrested/delayed (H). Embryos shown in B, E, F, G and H were all observed in sliques with cotyledon staged embryos. Size bar = 10 µm. (PDF) [file pgen.1004497.s005.pdf]
