# Supplementary material for: Arabidopsis thaliana WAPL Is Essential for the Prophase Removal of Cohesin during Meiosis
Source: PLoS Genet. 2014 Jul 17;10(7):e1004497. doi: 10.1371/journal.pgen.1004497 (PMC4102442; doi:10.1371/journal.pgen.1004497)
Supplement: Table S1 — Primers used in this study. Sequences of primers used in this study are shown. (PDF) [file pgen.1004497.s006.pdf]

Primers used in this study:

WAPL1.1 LP: CGACGCACTTTCCGTCC

WAPL1.1 RP: GAGCCAACGGTCGAGTA

WAPL1.2 LP: CGTCGCCGGACATCGAGCC

WAPL1.2 RP: GCCCTAATCCTCTTCAG

WAPL2 LP: GGTCTCAACAGCTTAACC

WAPL2 RP: GAGCGAACTTACGGCCGTCG

qPCR1 F : ACATCAATGTCGGGTTCTCA

qPCR1R : ACGCTAATCTCCCTGCAAAT

qPCR 2F: CACCAGATCTCCGCTTTACA

qPCR 2R: GTTTACCAGTAGCCCCAGGA

CTF7-F: CTCCTGATTTTCTGGGTTTCC

CTF7-R: TTCCAAACTGAATGGAGGTTG

LBb1.3 : ATTTTGCCGATTTCGGAAC
