# Supplementary material for: Arabidopsis thaliana WAPL Is Essential for the Prophase Removal of Cohesin during Meiosis
Source: PLoS Genet. 2014 Jul 17;10(7):e1004497. doi: 10.1371/journal.pgen.1004497 (PMC4102442; doi:10.1371/journal.pgen.1004497)
Supplement: Text S1 — Molecular characterization of Atwapl mutants. A description of the molecular analysis of the T-DNA insertion sites associated with AtWAPL1 and AtWAPL2 along with the corresponding genes is provided. (DOCX) [file pgen.1004497.s007.docx]

**Supplemental Text**

Two lines were characterized for *AtWAPL1* (*Atwapl1.1* (Salk_076791 and *AtWAPL1.2* (Salk_108385), and one line for *AtWAPL2* (Salk_127445). The *Atwapl1.2* and *Atwapl2* T-DNA insertions are in the first exon and intron, respectively, while the *Atwapl1.1* insert is located in intron 6. Plants homozygous for *Atwapl1.1*, *Atwapl1.2* and *Atwapl2* were selected by PCR genotyping using a combination of gene-specific and T-DNA primers. Primer combinations used for genotyping studies are: *Atwapl1.1*: *WAPL1.1RP* (5’-GAGCCAACGGTCGAGTA-3’) and *LBb1.3* (5’-ATTTTGCCGATTTCGGAAC-3’) and *WAPL1.1RP* with *WAPL1.1 LP* (5’ CGACGCACTTTCCGTCC-3’) to amplify the wild type allele; Atwapl1.2: *WAPL1.2 RP* (5’-GCCCTAATCCTCTTCAG-3’) and *LBb1.3* (5’-ATTTTGCCGATTTCGGAAC-3’) and *WAPL1.2 LP*: (5’-CGTCGCCGGACATCGAGCC-3’) and *WAPL1.2 RP* for the wild type allele; Atwapl2: *WAPL2 RP*: (5’-GAGCGAACTTACGGCCGTCG-3’) and *LBb1.3* : (5’-ATTTTGCCGATTTCGGAAC-3’) and *WAPL2 RP* with *WAPL2 LP*: (5’-GGTCTCAACAGCTTAACC-3’) for the wild type allele. In the *Atwapl1.1wapl2 ctf7-1* triple mutant the *Atctft7-1* (At4g31400 Salk_059500) T-DNA insertion site were screened with using *CTF7-R*: (5’-TTCCAAACTGAATGGAGGTTG-3’) and *LBb1.3* : (5’-ATTTTGCCGATTTCGGAAC-3’) wild the wild type allele are amplified with *CTF7-F*: (5’-CTCCTGATTTTCTGGGTTTCC-3’) and *CTF7-R*. Fragments corresponding to the T-DNA insertion sites were isolated and confirmed by DNA sequencing.

Primer combinations used for qPCR were as follows: *Atwapl1.1*: *qPCR1 F* (5’-ACATCAATGTCGGGTTCTCA-3’) & *qPCR1R* (5’-ACGCTAATCTCCCTGCAAAT-3’), for *Atwapl1.2:* *qPCR1 F* (5’-ACATCAATGTCGGGTTCTCA-3’) & *qPCR1R* (5’-ACGCTAATCTCCCTGCAAAT-3’) and for *Atwapl2* : *qPCR 2F*: (5’-CACCAGATCTCCGCTTTACA-3’) & *qPCR 2R*: (5’-GTTTACCAGTAGCCCCAGGA.
